# The gut microbiota of Indigenous populations in the context of dietary westernization: a systematic review and meta-analysis

**DOI:** 10.3389/fnut.2025.1652598

**Published:** 2025-10-22

**Authors:** Camille Daunizeau, Maximilien Franck, Amélie Boutin, Marianne Ruel, Natalia Poliakova, Pierre Ayotte, Richard Bélanger

**Affiliations:** 1 Population Health and Optimal Health Practices Research Unit, CHU de Québec Research Centre, Quebec city, QC, Canada; 2 Research Center on Aging, Faculty of Medicine and Health Sciences, Université de Sherbrooke, Sherbrooke, QC, Canada; 3 Reproduction, Mother and Youth Health Unit, CHU de Québec Research Centre, Quebec city, QC, Canada; 4 Department of Pediatrics, Faculty of Medicine, Université Laval, Quebec city, QC, Canada; 5 Library, Université Laval, Quebec city, QC, Canada

**Keywords:** traditional diet, westernized diet, gut microbiota, Indigenous populations, systematic review, meta-analysis, observational studies

## Abstract

**Background:**

Indigenous populations worldwide are undergoing dietary transitions from traditional patterns toward westernized diets, influencing gut microbiota diversity and composition, with potential implications for health.

**Objective:**

This systematic review and meta-analysis aimed to compare gut microbiota diversity and composition among Indigenous populations following traditional versus westernized dietary patterns.

**Methods:**

A comprehensive literature search was conducted in March 2024 and updated on February 25, 2025 across databases, including All Ovid MEDLINE®, Embase, Web of Science, CAB Abstracts, and Food Science and Technology Abstracts, along with searches of gray literature sources. Eligibility criteria included observational studies comparing gut microbiota diversity and composition between traditional and westernized diets among healthy Indigenous adults (≥16 years) without chronic diseases. Two reviewers independently performed study selection, data extraction, and risk of bias assessment using the ROBINS-E tool. Data were synthesized using random effects models, specifically applying the restricted maximum likelihood estimator to calculate between-study variance (τ^2^).

**Results:**

Of 19,836 articles identified, nine studies (*N* = 657 participants) met inclusion criteria. Traditional diets tended to be associated with higher microbial diversity, although results varied across diversity metrics and studies. Shannon diversity was higher in traditional groups, but this difference was not statistically significant (standardized mean differences = 0.67; 95% CI: −0.26 to 1.60; I^2^ = 92.9%). Other diversity indices (Chao1, Simpson, observed species richness) did not show clear differences between diet groups. Descriptive taxonomic analyses also revealed substantial heterogeneity across populations, reflecting the context-specificity of microbiota differences between traditional and westernized groups. Nonetheless, most westernized groups exhibited a higher Firmicutes/Bacteroidetes ratio at the phylum level and a lower *Prevotella*/*Bacteroides* ratio at the genus level.

**Conclusion:**

The observed heterogeneity likely reflects methodological differences, ecological variability, and the diversity of traditional diets and varying patterns of dietary transition. Longitudinal research is needed to better understand how dietary transitions affect gut microbiota over time in Indigenous populations.

**Systematic review registration:**

https://www.crd.york.ac.uk/PROSPERO/view/CRD42024597804.

## 1 Introduction

Humans are niche modifiers, continually changing their environments (1, 2). The Industrial Revolution marked an unprecedented shift in the human exposome—the totality of life-course environmental exposures (3)—profoundly influencing health patterns and directly contributing to the contemporary epidemic of chronic non-communicable diseases (CNCDs) in industrialized populations (4). Conversely, CNCDs are either rare or significantly reduced in certain Indigenous populations living a traditional lifestyle, such as hunter-gatherers, suggesting that these diseases emerge predominantly from environmental mismatches inherent to industrialized lifestyles (5–11).

The gut microbiota, a complex and dynamic ecosystem of microorganisms in the gastrointestinal tract, is essential for various physiological functions, including resistance to pathogen colonization, digestion, metabolism, immune function, and behavior (12–15). The typical dietary patterns of industrialized populations, known as the “Western diet,” are high in calorie density, animal protein/fat (saturated and omega-6 fatty acids, poor in omega-3), and sugar, while low in vitamins, minerals, fiber and phytochemicals from plant-based foods (16–18). Diet being a major driver of gut microbiota composition (19, 20), adhering to either westernized or traditional dietary patterns could have far-reaching implications for microbial balance and overall health. Accordingly, accumulating evidence indicates that adherence to westernized dietary patterns alters gut microbiota composition, typically reducing microbial diversity, which disrupts metabolic and immune functions, contributing to CNCDs (12, 21–24). Moreover, studies have shown that the gut bacterial composition of Indigenous populations differs significantly from that of industrialized populations, largely due to differences in their diets (25–31). Traditional diets are characterized by balanced macro- and micronutrient intake, limited refined carbohydrates, and abundant unsaturated fats, fibers, and phytochemicals (from plant-based foods) (7, 32–34). These diets vary considerably, spanning from subsistence diets, like those of the Yanomami (35) or the Tsimane (36), both from the Amazon, to agriculturally based diets (37), like that of the Caliata in current Ecuador (38). While most agriculturally based traditional diets are largely plant-based, the majority of hunter-gatherer traditional diets are predominantly animal-based (in terms of caloric intake) (33, 39).

Due to urbanization and integration into market economies, many Indigenous populations are adopting westernized dietary patterns, but at varying rates (40–44). Most existing knowledge on microbial differences between dietary patterns commonly labeled as “traditional” (e.g., Mediterranean) and westernized is derived from studies conducted in industrialized populations. Moreover, most of these populations had already begun transitioning toward industrialized lifestyles and westernized diets before the gut microbiota became a subject of scientific investigation. For these reasons, current knowledge may not be generalizable to Indigenous populations, whose dietary baselines and trajectories of transition differ substantially. Although interest in the gut microbiota of traditional populations is growing (25–31), there is a lack of systematic evidence on how diverse traditional-to-Western dietary transitions affect microbiota diversity and composition across Indigenous populations. This systematic review and meta-analysis examines observational studies comparing gut microbiota composition and diversity between Indigenous adults adhering to traditional versus westernized dietary patterns to evaluate the impact of dietary transitions on the gut microbiota.

## 2 Methods

This systematic review adheres to the Preferred Reporting Items for Systematic Reviews and Meta-Analyses Protocols (PRISMA-P) guidelines and to the methodological recommendations of the Cochrane Handbook for Systematic Reviews of Interventions (45, 46). The protocol for this review was registered in PROSPERO (registration number: CRD42024597804) on October 7, 2024, after beginning the initial database search in March 2024.

### 2.1 Eligibility criteria

#### 2.1.1 Types of studies

Due to ethical and logistical challenges associated with conducting interventional studies in Indigenous communities, and given that the existing literature is predominantly observational, we decided *a priori* to include only observational studies (cohort, case–control, and cross-sectional designs) comparing gut microbiota composition and diversity between individuals adhering to traditional versus westernized dietary patterns in Indigenous communities.

Eligible studies included:

1. Studies with a primary objective of comparing the effects of traditional versus westernized diets on gut microbiota composition within the same Indigenous population.
2. Studies whose primary objective was not explicitly focused on diet but that provided sufficiently detailed descriptions of dietary practices in Indigenous communities (e.g., through structured questionnaires, interviews, or ethnographic documentation) and allowed for comparison between traditional and westernized dietary patterns.
3. Studies comparing gut microbiota composition between two or more geographically close populations (e.g., within the same country or region), with documented differences in dietary patterns (traditional vs. westernized), including those without formal dietary data collection, provided that descriptive information was sufficiently detailed.
4. Studies comparing gut microbiota composition between two geographically distinct populations, described by the original authors as sharing a common ethnic or genetic background, with documented differences in dietary patterns (traditional vs. westernized).

Eligibility for quantitative synthesis (pre-specified pooling rules):

We prespecified that meta-analysis would be limited to studies reporting alpha-diversity, with extractable means and standard deviations or summary statistics convertible to means and SDs (see Data extraction). Studies reporting other diversity metrics or insufficient summary data were included in the review but synthesized narratively. We pooled diversity metrics or taxa only when ≥ 3 independent studies reported the same metric or taxon with compatible definitions and scales (relative abundance as % or proportion, or convertible thereto); all other metrics or taxa were summarized descriptively.

#### 2.1.2 Participant characteristics

This systematic review and meta-analysis included healthy young people or adults (16 and older) from Indigenous communities who do not have reported chronic diseases. Studies conducted among pregnant women exclusively were non-eligible. Comparator groups were not necessarily Indigenous but had to be either geographically proximate (same country or region) with a westernized/industrialized lifestyle, or, if from a different geographic area, ethnically similar to the Indigenous group under study.

Indigenous communities are defined according to the United Nations Declaration on the Rights of Indigenous Peoples (UNDRIP) (47, 48) as “those which, having a historical continuity with pre-invasion and pre-colonial societies that developed on their territories, consider themselves distinct from other sectors of the societies now prevailing on those territories, or parts of them. They form at present non-dominant sectors of society and are determined to preserve, develop and transmit to future generations their ancestral territories, and their ethnic identity, as the basis of their continued existence as peoples, in accordance with their own cultural patterns, social institutions and legal system.”

Although the UNDRIP definition served as a conceptual reference for this review, it was not applied as a strict inclusion criterion. Instead, studies were included if the participants were described as “Indigenous” or “tribal” by study authors, or if their communities were recognized as Indigenous in authoritative sources such as the UN, UNESCO, the International Work Group for Indigenous Affairs (IWGIA), or in peer-reviewed anthropological literature. In addition, certain populations not formally recognized as Indigenous, but whose lifestyles aligned with the conceptual framework of this review (e.g., traditional subsistence practices, ancestral diets, ecological rootedness, and ongoing transitions in dietary patterns) were also included due to their strong relevance to the objectives of this review. The identification of these populations was based on detailed descriptions provided in the included studies and, when necessary, supplemented by anthropological sources documenting their subsistence strategies and cultural practices.

#### 2.1.3 Dietary classification

Dietary classification was based on an assessment of descriptive information provided in each study. Although explicit use of the terms “traditional” or “westernized” by the original authors was not required, studies had to provide sufficiently clear descriptions of participants’ dietary habits to allow for consistent categorization. Studies lacking adequate dietary description were excluded from this systematic review and meta-analysis.

##### 2.1.3.1 Exposure group

Eligible studies compared gut microbiota composition and diversity between individuals adhering to traditional versus less traditional or westernized dietary patterns within Indigenous communities. In the present study, we define “traditional diets” as the customary dietary practices historically followed by the Indigenous populations under study. These diets are shaped by long-standing ecological, cultural, and subsistence patterns and typically consist of locally available, minimally processed foods prepared using traditional methods. While the content of these diets varies across groups—ranging from hunter-gatherer to horticultural and agrarian food systems—they are unified by their continuity with pre-industrial foodways and their central role in cultural identity and social organization (40, 49, 50).

##### 2.1.3.2 Comparator group

Westernized diets refer to dietary patterns that emerged following industrialization in the 19th and 20th centuries, marked by a growing reliance on processed and ultra-processed foods (16, 51). These foods are characterized by the addition of natural or artificial compounds during industrial processing, such as refined cereal grains, sugars, salt, vegetable oils and food additives (e.g., colorants, emulsifiers, artificial sweeteners, preservatives) (16, 51). Westernized dietary patterns are commonly followed in industrialized populations and are typically high in calories, rich in animal proteins, saturated fats, and monosaccharides, while being low in micronutrients and non-digestible components originating from plants (fibers and phytochemicals) (16, 17, 51).

For inclusion in this review, studies had to provide sufficiently clear descriptions of participants’ dietary habits to enable consistent classification of diets as either “traditional” or “westernized.” Groups exposed to westernized diets were identified either through author-reported labels (e.g., “urbanized,” “westernized”) or, in the absence of such labels, based on reported consumption of market-based or processed foods.

#### 2.1.4 Outcome measures

The primary outcome is between-group differences (traditional versus westernized diets) in alpha-diversity of the gut microbiota, assessed through 16S ribosomal RNA sequencing, shotgun metagenomics or other established microbiota analysis techniques.

When data are available, we analyze the relative abundance of specific microbial taxa (phyla, families, genera, etc.), comparing the mean abundances between the group following a traditional diet (exposure) and the group following a westernized diet (comparator). The definition of the research question using the PECOS criteria is presented in Table 1.

**Table 1:** Definition of the research question using PECOS criteria.

Terms of PECOS	Definition of terms
Population	Healthy individuals aged 16 and older from Indigenous populations, as defined by the United Nation; excluding pregnant women and participants with chronic diseases.
Exposure	Adherence to a traditional diet, characterized by locally and seasonally available minimally processed foods and cultural culinary practices emphasizing fresh, traditional foods.
Comparator	Adherence to a westernized diet, characterized by high consumption of processed and ultra-processed foods rich in calories, animal proteins, saturated fats, refined sugars, and low in fiber and micronutrients.
Outcome	Differences in gut microbiota alpha-diversity and relative abundances of specific bacterial taxa (phyla, families, genera, etc.) between exposure and comparator groups, assessed through 16S rRNA sequencing, shotgun metagenomics, or other established methods.
Study population	Observational studies (cohort, cross-sectional, case–control).

### 2.2 Data sources and search strategy

A comprehensive search strategy initially performed on March 18, 2024, and updated on February 25, 2025, was conducted across multiple databases, including All Ovid MEDLINE®, embase.com (Embase, Medline, Preprints, PubMed-not-Medline), Web of Science (core collection as provided by Université Laval; Editions = SCI-EXPANDED, SSCI, AHCI, CPCI-S, CPCI-SSH, BKCI-S, BKCI-SSH, ESCI, CCR-EXPANDED, IC), CAB Abstracts (OvidSP) and Food Science and Technology Abstracts (OvidSP). The search strategy was developed and implemented in collaboration with an information specialist and it incorporated both free-text and controlled vocabulary terms related to “Indigenous populations,” “traditional food,” and “gut microbiota composition.” Gray literature was identified through searches in the Conference Proceedings Citation Index via Web of Science and the preprint indexes in Embase and Medline.

The detailed research strategy for Medline/OVID is presented in Supplementary Table 1.

### 2.3 Risk of bias assessment

We evaluated the risk of bias in the included studies using the ROBINS-E (Risk Of Bias In Non-randomized Studies of Exposures) criteria. Based on the latest version of ROBINS-E, the most relevant signaling questions for assessing the risk of bias in the eligible studies were retained for bias analysis. Each signaling question could have responses of: No (N), Probably No (PN), Yes (Y), and Probably Yes (PY). The signaling questions belonged to different domains of bias, namely: (D1) confounding, (D2) exposure measurement, (D3) participant selection, (D4) post-exposure interventions, (D5) missing data, (D6) outcome measurements, (D7) selective reporting.

Based on the ROBINS-E algorithms presented for each domain of bias, and depending on the responses given to the signaling questions, the domains of bias could be classified from low risk, through some concerns, to very high risk. To evaluate the overall risk of bias for each study, we aggregated the ratings from all domains. Specifically, if any domain was rated as having a “high risk of bias,” the overall risk of bias for that study was also classified as “high risk of bias.” For further details, please refer to the original publication by the ROBINS-E Development Group (45).

### 2.4 Data extraction and preparation

References were managed using Covidence, a specialized software for study selection and data extraction. Duplicates were automatically removed by Covidence, and two independent reviewers (C.D. and M.F.) screened all studies by assessing their titles and abstracts. Potentially relevant studies were then evaluated for inclusion by the same reviewers through a full-text review. Any conflicts were resolved by discussion. Data extraction was conducted using a standardized extraction form developed specifically for this review. The form was pilot-tested on a subset of 3 articles to ensure consistency and comprehensiveness before full extraction. The extraction form captured the following information: study information (publication date, study design, objectives, and hypotheses); eligibility assessment (eligibility criteria reported in the study, whether the study was included or not in the review, and reasons for inclusion or exclusion); study characteristics (funding sources, declared conflicts of interest, participant eligibility criteria, sample size, and primary/secondary outcomes); participant flow (number of eligible individuals, number excluded, total number of participants included); participant characteristics (mean age and BMI in exposure and comparator groups, health status, and relevant lifestyle factors); exposure and comparator details (method of dietary assessment and description of dietary patterns for both exposure and comparator groups); outcome description (methods used to assess gut microbiota composition, means and SDs of alpha and beta diversity measures in each group); statistical analysis (statistical models and adjustment variables used, if available) and study conclusions (key conclusions drawn by the study authors).

For transparency and consistency with PRISMA 2020, we recorded for each included study (i) the total sample size initially enrolled by the primary investigators and (ii) the number of sequenced participants who met this review’s eligibility criteria. Sequenced participants denotes individuals for whom gut-microbiota data were successfully generated (16S rRNA or shotgun metagenomics) and retained after applying this review’s eligibility criteria (e.g., age ≥ 16 y, healthy/non-pregnant status as defined). By contrast, the total sample size initially enrolled may include individuals for whom no microbiota data were available (for example, stool not collected, low DNA yield, failed library or sequencing run) or who otherwise did not meet our eligibility criteria. Only sequenced participants contributed to quantitative syntheses. For studies presenting data exclusively via boxplots, individual-level or group-level summary statistics (means and standard deviations) were extracted using PlotDigitizer software (available online at https://plotdigitizer.com). Means and standard deviations were estimated from available medians, minimums, maximums, and sample sizes (N) using the method described by Hozo (52). When raw taxonomic count data (e.g., OTU tables) were available but individual-level diversity or composition data were not, relative abundances were computed by normalizing taxon counts to total reads per sample. When individual-level alpha-diversity data were available across multiple time points, we retained baseline values rather than computing averages over time. In cases where multiple comparator groups were present within a study, groups with similar characteristics were combined to increase sample size and analytical power. Sensitivity analyses were performed using the original, unpooled groups to assess the robustness of the findings. Combined means and standard deviations for merged groups were calculated using established formulas for pooling summary statistics, such as the one described by Higgins (45). Specific details related to data extraction and processing for each study are provided in Supplementary material.

### 2.5 Statistical analysis

Analyses were conducted in R using the *metafor* package (version 2024.12.1; (53)). Data were synthesized using standardized mean differences (SMD) adapted for heteroscedastic population variances as the effect size measure, for both alpha-diversity and microbial taxa abundances. A random effects model was used to analyze the data, with the restricted maximum likelihood (REML) estimator selected to estimate the between-study variance (τ^2^). REML was selected for its ability to provide less biased estimates of heterogeneity and is known to be reasonably robust under non-normal conditions (54, 55). Sensitivity analyses included refitting models with the Sidik-Jonkman estimator (Supplementary Figure 1) and repeating the alpha-diversity meta-analyses after excluding studies that enrolled participants older than 65 years (Supplementary Figure 2). Finally, with fewer than 10 studies, the number of included studies was not sufficient to reliably assess publication bias using a funnel plot.

## 3 Results

### 3.1 Overview

**Figure 1:**
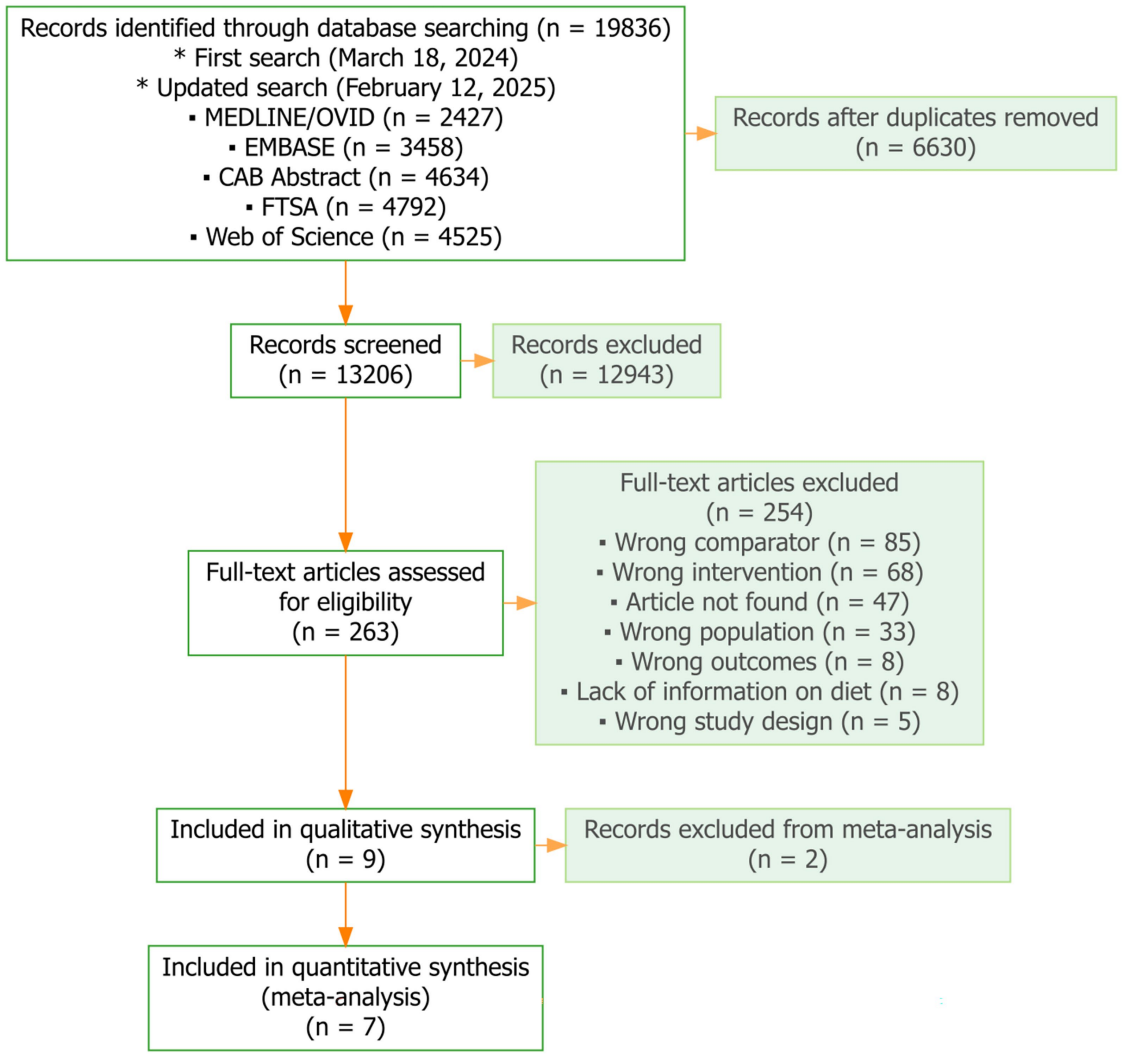
Flow diagram illustrating the identification, screening, eligibility assessment, and inclusion of studies. The diagram follows PRISMA (Preferred Reporting Items for Systematic Reviews and Meta-Analyses) guidelines. Nine studies were assessed and included in qualitative synthesis (*n* = 9), of which seven with comparable diversity index metrics were included in the quantitative synthesis (meta-analysis; *n* = 7), and two were excluded from the meta-analysis due to non-comparable metrics.

The search strategy yielded a total of 19,836 articles from six databases (Figure 1). After removing 6,630 duplicates, 13,206 articles were screened based on title and abstract. Of these, 263 articles were selected for full-text review. Among the 263 full-text articles assessed, 254 were excluded because they did not meet the eligibility criteria. Ultimately, nine studies met the inclusion criteria and were synthesized qualitatively; seven of these reported comparable alpha-diversity metrics and were included in the meta-analysis, and the remaining two, with non-comparable metrics, were summarized narratively.

### 3.2 Characteristics of the included studies

**Figure 2:**
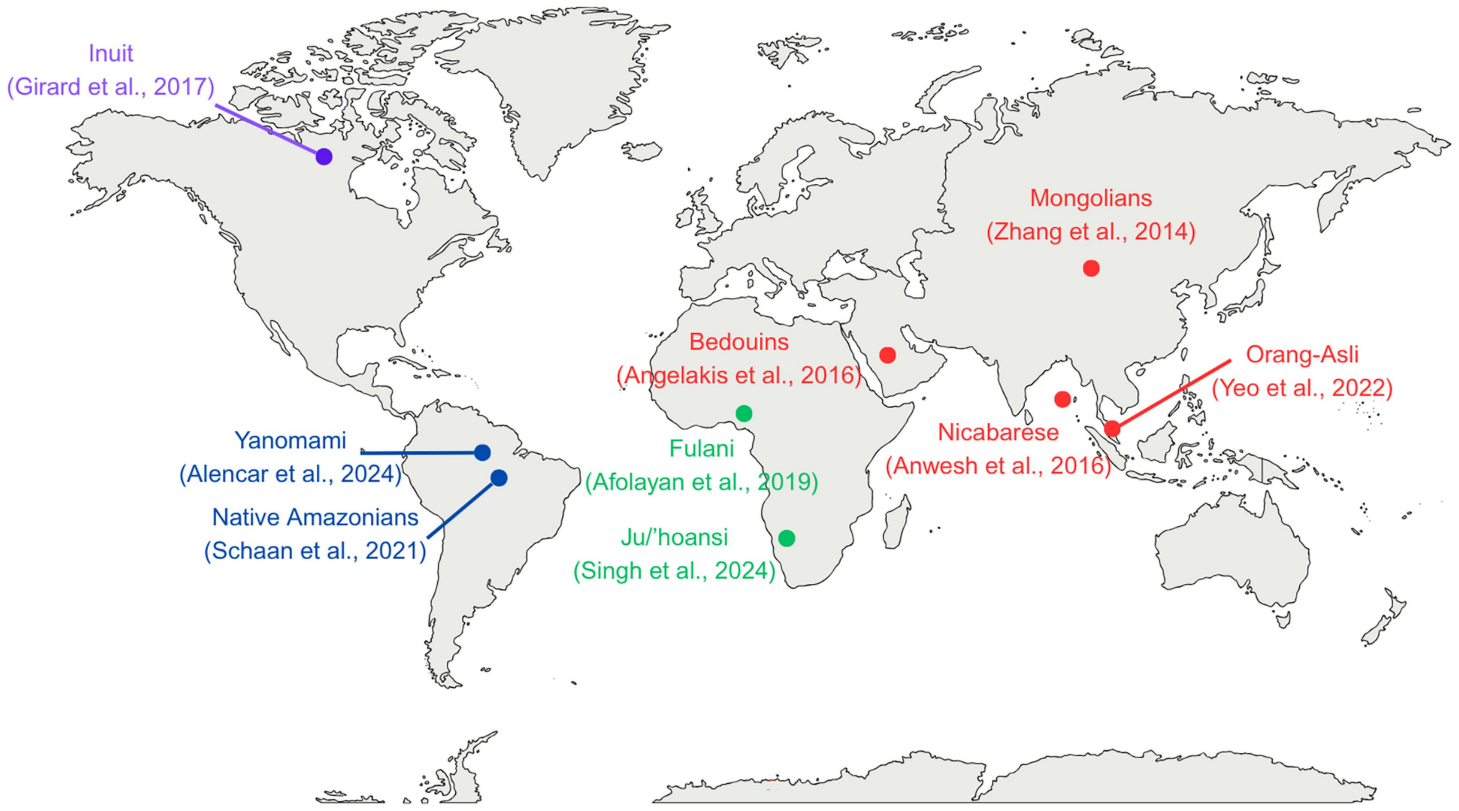
Map of communities included in the present systematic review and meta-analysis. Locations are approximate. Colors correspond to world regions as follows: violet for the Arctic region, red for Asia, green for Africa, and blue for South America.

Across the nine included studies, 768 individuals were enrolled in total, of whom 657 were successfully sequenced and met this review’s eligibility criteria. The participants came from various regions of the world: Africa (Nigeria (56); Namibia (57)), South America (Brazil (58, 59)), Asia [Malaysia (60); Mongolia (61); Saudi Arabia (62), India (63)], and the Arctic region [Canada (26)] (Figure 2). All included studies met the inclusion criteria, with participants either described as “Indigenous” or “tribal” by the study authors, recognized as such in international databases (e.g., UN, UNESCO, IWGIA) or identified in authoritative anthropological literature. One study conducted in Mongolia involved nomadic pastoralists from Khentii Province who were not officially recognized as Indigenous but whose way of life, characterized by traditional pastoralist subsistence, ancestral dietary practices, and a long-standing cultural relationship with the land, closely aligned with the conceptual framework of this review. Although these participants were not officially recognized as Indigenous in international sources, nomadic pastoralism remains a prevalent mode of subsistence in Mongolia, with approximately 30% of the population continuing to engage in this practice (64). The persistence of this lifestyle in rural areas, including typical pasture regions such as Khentii, supports the relevance of this population to the objectives of the review. A sensitivity analysis excluding this study was performed to ensure that its inclusion did not bias the overall findings (Supplementary Figure 3).

Dietary assessment methods varied across studies. Three studies used dietary questionnaires: an annual dietary habit questionnaire (26), a standardized questionnaire (62), and a food frequency questionnaire assessing long-term dietary intake (61). Three other studies relied on interviews to describe dietary habits (56, 58, 60), with one justifying this choice by the low literacy level of participants (60). Finally, three studies (57, 59, 63) inferred dietary patterns from lifestyle and community descriptions, without explicit mention of interviews or structured questionnaires. Traditional diets were generally composed of high-fiber, minimally processed foods (e.g., tubers, wild plants, fruits, fish, fermented foods) except for the traditional Inuit diet which was essentially animal-based (33, 40, 41), while westernized diets included refined carbohydrates, meats, dairy products, and industrially processed foods. Most studies used 16S rRNA gene sequencing to characterize the gut microbiota, while one study (57) used shotgun metagenomics. Alpha-diversity was assessed in all studies, mainly using Shannon and Chao1 indices. Taxonomic resolution typically reached the phylum or genus-level, and one study (57) included species-level analysis. The methodological characteristics of the studies are detailed in Table 2 and participant characteristics are summarized in Table 3.

**Table 2:** Characteristics of observational studies included in the meta-analysis (investigating the effect of traditional vs. westernized food consumption on the gut microbiota composition).

Authors	Country	Study design	Total sample size^1^	Dietary assessment method	Sequencing method	Platform used for sequencing	Bioinformatic analysis pipeline	Alpha-diversity metrics used	Lowest taxonomic level analyzed
Afolayan, A. O. (2019) (56)	Nigeria	Observational, cross-sectional	50	Verbal interviews	16S rRNA gene sequencing (V4 region)	Illumina MiSeq	QIIME (v1.9.1)	Evenness (Pielou’s measure of species evenness), observed species, Chao1 index, Shannon index, phylogenetic diversity (PD) whole tree, Simpson index	Genus-level
Alencar, R. M. (2024) (59)	Brazil	Observational, cross-sectional	30	Not assessed directly	16S rRNA gene (V1–V2 regions)	Ion-Torrent PGM	Mothur	ACE diversity indices, number of bacterial genera identified	Genus-level
Angelakis, E. (2016) (62)	Saudi Arabia	Observational, cross-sectional	28	Standardized questionnaire	16S rRNA gene sequencing (V3–V4 regions)	Illumina MiSeq	QIIME (v1.8.0)	Chao1 index, Shannon index, number of observed OTUs, number of observed bacterial genera	Genus-level
Anwesh, M. (2016) (63)	India	Observational, cross-sectional	60	Not assessed directly	16S rRNA gene sequencing (V3 region)	Illumina MiSeq	QIIME (v1.9.0)	Shannon index	Genus-level
Girard, C. (2017) (26)	Canada	Observational, cross-sectional	45	Food frequency questionnaire (over 1 year)	16S rRNA (V4 region)	Illumina MiSeq	QIIME (v1.8.0)	Shannon index, Simpson index, Chao1-estimated OTUs, observed OTUs, Fisher diversity index	Strain-level via olygotyping
Schaan, A. P. (2021) (58)	Brazil	Observational, cross-sectional	114	Individual dietary habits interviews	16S rRNA gene sequencing (V3–V4 region)	Illumina MiSeq	QIIME2	Number of observed species, Simpson, Chao1 and Shannon diversity metrics	Genus-level
Singh, H. (2024) (57)	Namibia	Observational, cross-sectional	161	Not assessed directly	Shotgun metagenomics	Illumina NovaSeq 6000	VEBA pipeline	Shannon index	Species-level
Yeo, L.-F. (2022) (60)	Malaysia	Observational, cross-sectional	216	Descriptive dietary recall interviews	16S rRNA (V3–V4 and V4 regions)	Illumina MiSeq	QIIME2 (v2021.4)	Microbial richness, Shannon index (evenness) and Pielou’s evenness	Genus-level
Zhang, J. (2014) (61)	Mongolia	Observational, longitudinal (5 seasonal time points)	64	Food frequency questionnaire (5 seasonal points)	16S rRNA gene sequencing (454 pyrosequencing)	454 GS FLX Titanium (Roche)	QIIME (v1.2.1)	Shannon, Simpson, Chao1, Observed species	Genus-level

^1^The “Total sample size” corresponds to the total number of participants initially included across eligible studies, prior to applying the meta-analysis specific inclusion criteria.

**Table 3:** Characteristics of participants in included studies.

Authors	Population group	No. of sequenced participants^1^	Female/Male (*n*)	Age (years): mean ± SD, range or category	BMI (kg/m²): mean ± SD, category or prevalence	Health status	Country or region of participants	Type of diet	Additional lifestyle factors
Afolayan, A. O. (2019) (56)									
Exposure	Fulani: nomadic pastoralists	9	4/5	Adults (16–65)	N/A	Healthy	Nigeria (Pabaman-shanu village)	Traditional, high-fiber diet based on local grains, fermented drinks, and vegetables, with minimal processed foods.	Nomads, living in a rural environment. No access to medical supply.
Comparator	Jarawa: semi-urbanized Nigerian population	15	8/7	Adults (16–65)	N/A	Healthy	Nigeria (Lamingo, Jos)	More westernized diet including processed foods, refined grains, bread, and pasta, with greater exposure to industrial products. Eat meat regularly.	Living in a city, access to medical supply, processed foods and safe water.
Alencar, R. M. (2024) (59)									
Exposure	Yanomami: Indigenous, hunter-gatherers	10	6/4	Adults (35–65)	N/A	Healthy	Brazil, (Roraima and Amazonas states)	High-fiber plant-based diet from the forest: roots, seeds, fruits, fish, occasionally meat and little to no processed food.	Hunter-gatherer society from the Amazon, undergoing a transition to urbanization.
Comparator	Manaus: urban Brazilians	7	5/2	Adults (35–65)	N/A	Healthy	Manaus, Amazonas	Mixed with consumption of industrialized foods.	Urban: access to health care, medicine, markets.
Angelakis, E. (2016) (62)									
Exposure	Bedouins	11	2/8	Adults	N/A	Healthy	Rural Saudi Arabia	Vegetables, fruit, homemade fermented dairy products, chicken, rice.	Pastoralist lifestyle.
Comparator	Urban Saudis	18	0/18	Adults	N/A	Healthy	Jeddah, Saudi Arabia	Limited vegetables/fruits, processed snacks, fast food (shawarma, hamburger, pizza, fried chicken), carbonated beverages.	Have recently adopted a drastically changed lifestyle with poor dietary diversity and little to no fruit or vegetable intake.
Anwesh, M. (2016) (63)									
Exposure	Nicobarese remote	36	N/A	Adults (20–73)	21/60 overweight	Healthy (30% hypertensive)	Remote villages of Nancowry group of Islands, India	Whole-grains, wild or cultivated tubers, fruits, fermented foods, marine produce, meat of wild boar or domestic pigs and poultry. Minimal use of tinned foods and beverages.	Minimal access to goods and opportunities for social interaction with other communities. Use of minimal therapeutic drugs and dependence on traditional medicine.
Comparator 1	Nicobarese rural	12	N/A	Adults (20–73)	21/60 overweight	Healthy (30% hypertensive)	Rural areas of Nancowry group of Islands, India	Mixed subsistence: cereals and grains (from grocery stores or produced by their farms), poultry, domesticated pigs and chicken.	Access to goods and opportunities for social interaction with non-tribal communities. Farming practices. Access to health center.
Comparator 2	Nicobarese urban	12	N/A	Adults (20–73)	21/60 overweight	Healthy (30% hypertensive)	Residing in Port Blair town, Andaman and Nicobar Islands, India	Rely predominantly on imported cereals, pulses, and locally purchased meat products, with regular consumption of processed and canned foods.	Have easy access to diverse goods, services, and social interactions with non-tribal communities; Access to pharmacies, hospitals, and modern healthcare facilities.
Girard, C. (2017) (26)									
Exposure	Inuit (Inuit diet)	16	7/13	Adults (42 ± 16)	28.25 ± 7.42	Healthy	Nunavut, Canada	Inuit diet: traditional meats (caribou, seal, whale, fish); little plant-derived food; rich in animal protein, source of vitamins, minerals, and micronutrients.	Remote, subsistence-based hunting and fishing communities. Underwent a rapid dietary shift from traditional foods to processed.
Comparator	Inuit (Western diet)	3	2/1	Adults (42 ± 16)	22.85 ± 3.19	Healthy	Nunavut, Canada	Western diet: processed foods, lower micronutrients intake.	Higher food diversity.
Schaan, A. P. (2021) (58)									
Exposure	Xikrin: Indigenous (Bacajá Xikrin)	12	N/A	Adults (20-60)	N/A	Healthy (highest protozoa and helminth prevalence)	Brazilian Amazon, Trincheira-Bacajá Indigenous Territory	High-fiber traditional diet: subsistence agriculture (sweet potatoes, cassava, corn, pumpkin and bananas), small game hunting, fishing and gathering of nuts and fruits. Great food variety.	Very remote. Rely on subsistence farming. Access to the Xikrin group is difficult due to the dense Amazon rainforest and poor road infrastructure.
Comparator 1	Suruí: Indigenous (Suruí-Aikewara)	16	N/A	Adults (20-60)	N/A	Healthy	Brazilian Amazon, Sororó Indigenous Territory	Rice cultivation, small game hunting. Progressively shifting from subsistence farming (cassava, sweet potatoes) to increased consumption of industrialized foods (frozen poultry, sugar, dairy, crackers).	Semi-remote, increasing access to westernized food. The Suruí are accessible by land.
Comparator 2	Tupaiú: Indigenous (multiethnic emergent group)	16	N/A	Adults (20-60)	N/A	Healthy	Tapajós-Arapiuns Extractive Reserve, Brazilian Amazon	Diet dependent on fishing, fruit harvesting, cassava root, small game. Some industrial products.	Access to their territory only by boat/helicopter. More mixed and transitioning population.
Comparator 3	Belém: urban residents of Belém	25	N/A	Adults (20-60)	N/A	Healthy	Belém, capital of Pará State, Brazilian Amazon	Mixed diet: mainly rice, beans, animal protein, manioc flour, dairy products and industrialized foods.	Recruited in the Federal University of Pará (university students, faculty members and surrounding neighborhoods).
Singh, H. (2024) (57)									
Exposure	Ju/΄hoansi: Indigenous, hunter-gatherers	55	33/28	Adults	N/A	Healthy	Kalahari Desert, Namibia	Plant-centric diet rich in nuts, fruits, roots, leafy greens (~105 wild edible plants), very limited meat consumption.	Foraging and hunting lifestyle. Adapted to seasonal resource scarcity by combining traditional foraging with market-based strategies.
Comparator 1	Bantu: agropastoral community	16	N/A	Adults	N/A	Healthy	Namibia, geographically close to Ju/'hoansi	Mixed subsistence: plant-based foods, farming, market-based strategies.	Agropastoralist population, slightly more market-based integration than Ju/‘hoansi.
Comparator 2	Healthy WU: African-descendant urban Trinidadians	90	N/A	Adults	N/A	Healthy	Trinidad (Caribbean)	Westernized: processed foods.	People of African descent from urban Trinidad.
Yeo, L.-F. (2022) (60)									
Exposure	Jehai: Indigenous Orang-Asli, Negrito subgroup	83	N/A	Adults (≥18)	25% obese	Healthy (33% with MetS)	Royal Belum Rainforest, Perak	Various leafy greens that grew wild in the jungle, along with fishing. Hunting less frequent.	Forest-dwelling hunter-gatherers; minimal access to store-bought foods.
Comparator 1	Temiar PP: rural Temiar	68	N/A	Adults (≥18)	21% obese	Healthy (54% with MetS)	Pos Piah village, Perak	Not fully detailed, but lower access to store-bought food than Temiar GM.	Rural, less remote than Jehai. Limited market access.
Comparator 2	Temiar GM: semi-urban Temiar	29	N/A	Adults (≥18)	21% obese	Healthy (54% with MetS)	Resettlement villages in Gua Musang, Kelantan	Mixed diet: store-bought food (rice, biscuit, canned food, bread, chicken, fish) and plants (sweet potato leaves).	Semi-urban, moderate land development around village. Occasional hunting of small mammals.
Comparator 3	Temuan: Urbanized Orang-Asli	34	N/A	Adults (≥18)	71% obese	Poorer cardiometabolic health	Bukit Lanjan, Selangor	Westernized diet: rice, bread, biscuits, fish, chicken, deficient in plant fiber, fast-food products.	Urban lifestyle.
Zhang, J. (2014) (61)									
Exposure	Khentii: Mongolians with nomadic lifestyle	12	N/A	Adults (36 ± 17)	21 ± 4	Healthy	Khentii Province, Mongolia	Typical Mongolian diet with seasonal variation: high and frequent consumption of fermented dairy, red meat, liquor. Low food diversity.	Traditional nomadic lifestyle, strong seasonal variation in diet (mostly meat in winter/spring, dairy products in summer/autumn).
Comparator 1	TUW: Semi-urban Mongolians	16	N/A	Adults (43 ± 12)	24 ± 5	Healthy	Suburbs of Ulan Bator, Mongolia	Intermediate diet diversity, moderate seasonal variation.	Have adopted an urban lifestyle. Moderate exposure to modernization.
Comparator 2	Ulan Bator: Urban Mongolians	36	N/A	Adults (29 ± 9)	20 ± 4	Healthy	Ulan Bator, Mongolia	Westernized diet: limited changes throughout the year.	Urban environment.

^1^The “No. of sequenced participants” represents the number of individuals who were successfully sequenced and met the eligibility criteria for our systematic review and meta-analysis. ^2^Not fully sequenced. N/A, Not Available.

### 3.3 Risk of bias assessment

**Figure 3:**
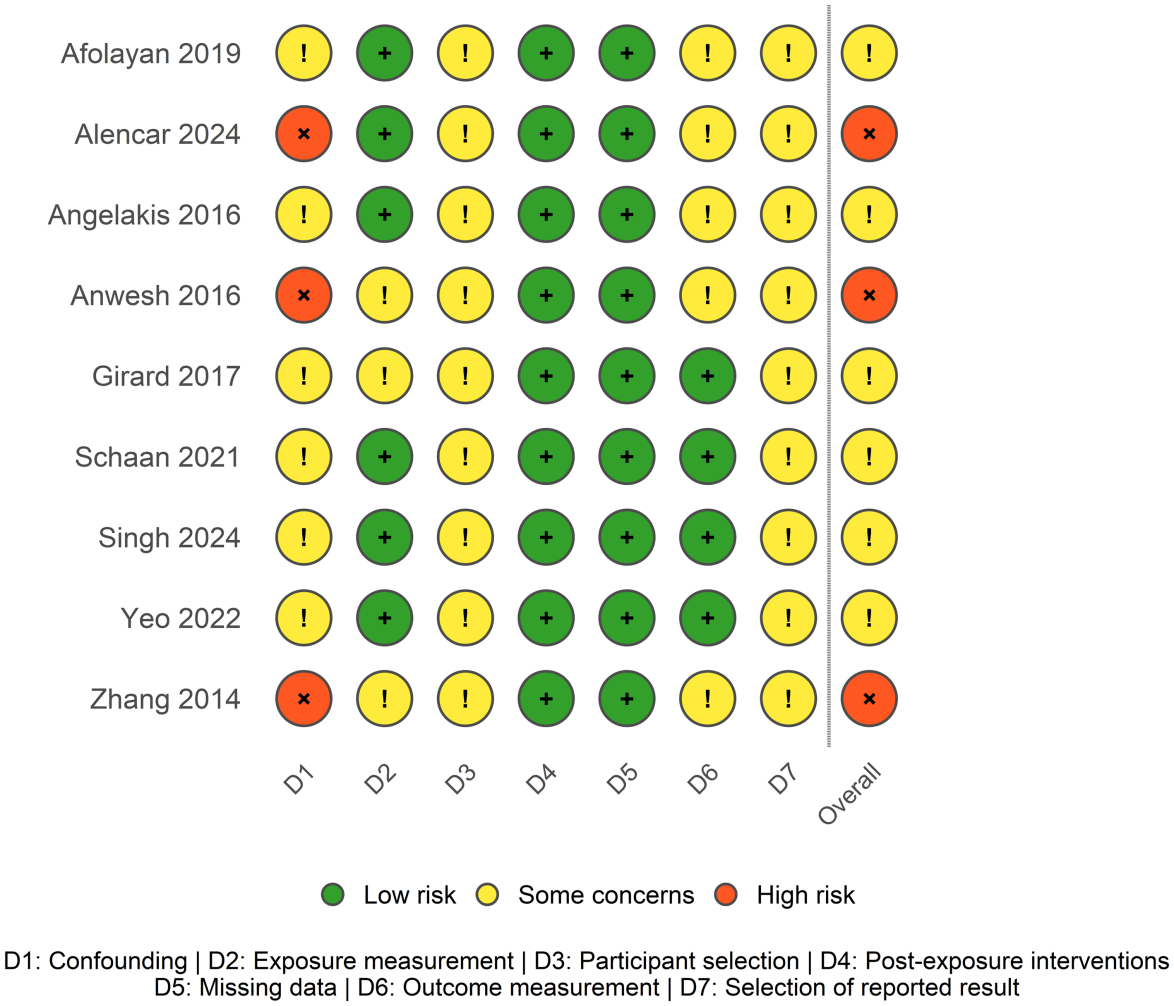
Risk of bias assessment across included studies.

Among the nine studies included in our systematic review and meta-analysis, three (59, 61, 63) were rated as having a high overall risk of bias, primarily due to issues in the confounding domain (Figure 3). Zhang (61) reported excluding participants with a history of gastrointestinal disease, but did not account for other potential confounding factors such as socio-economic status, medication use, or antibiotic exposure and Alencar (59) and Anwesh (63) did not mention any exclusion criteria. The remaining six studies were judged at moderate risk of bias overall, with some concerns across several domains, particularly in the domains of participant selection (D3), outcome measurement (D6), and selection of reported results (D7).

Due to the observational design of the included studies, there was no randomization in the selection of participants. All studies relied on voluntary participation, which may introduce selection bias. Angelakis (62) included almost exclusively male participants, limiting the representativeness of the study sample. Girard (26) was the only study to report a coverage rate, indicating that 26 Nunavut volunteers represented approximately 18% of the local adult population, suggesting potential recruitment bias. This relatively low rate highlights a common challenge in studies conducted within Indigenous communities, where the number of participants is often limited (geographic remoteness, language barriers, and the necessity of culturally appropriate consent involving community leaders). As a result, most studies included in this meta-analysis involved small sample sizes, which may reduce statistical power and limit the precision and generalizability of the pooled estimates.

In all included studies, exposure and comparator groups were geographically proximate, except for Singh (57), who compared the Ju/'hoansi of Namibia with Western-Urban individuals from Trinidad. However, the authors explicitly justified this choice by highlighting the genetic similarity between these populations, as the Trinidadian cohort comprised individuals of African descent sharing significant genetic ancestry with the Ju/'hoansi. In the exposure measurement domain, Girard (26) and Zhang (61) were considered at moderate risk, as they relied on food frequency questionnaires. In particular, the food frequency questionnaire used in Girard (26) covered a one-year dietary recall period, which may introduce recall bias and temporal mismatch between dietary exposure and microbiota sampling. Across the nine studies included in this review, bioinformatic quality control procedures—such as filtering low-quality reads, removing chimeras, and trimming sequences—were generally well implemented. However, only two studies (57, 60) reported the use of technical quality controls, such as mock communities or extraction blanks, which are important for detecting contamination and validating pipeline performance.

### 3.4 Associations between dietary westernization and gut microbiota diversity and composition

**Figure 4:**
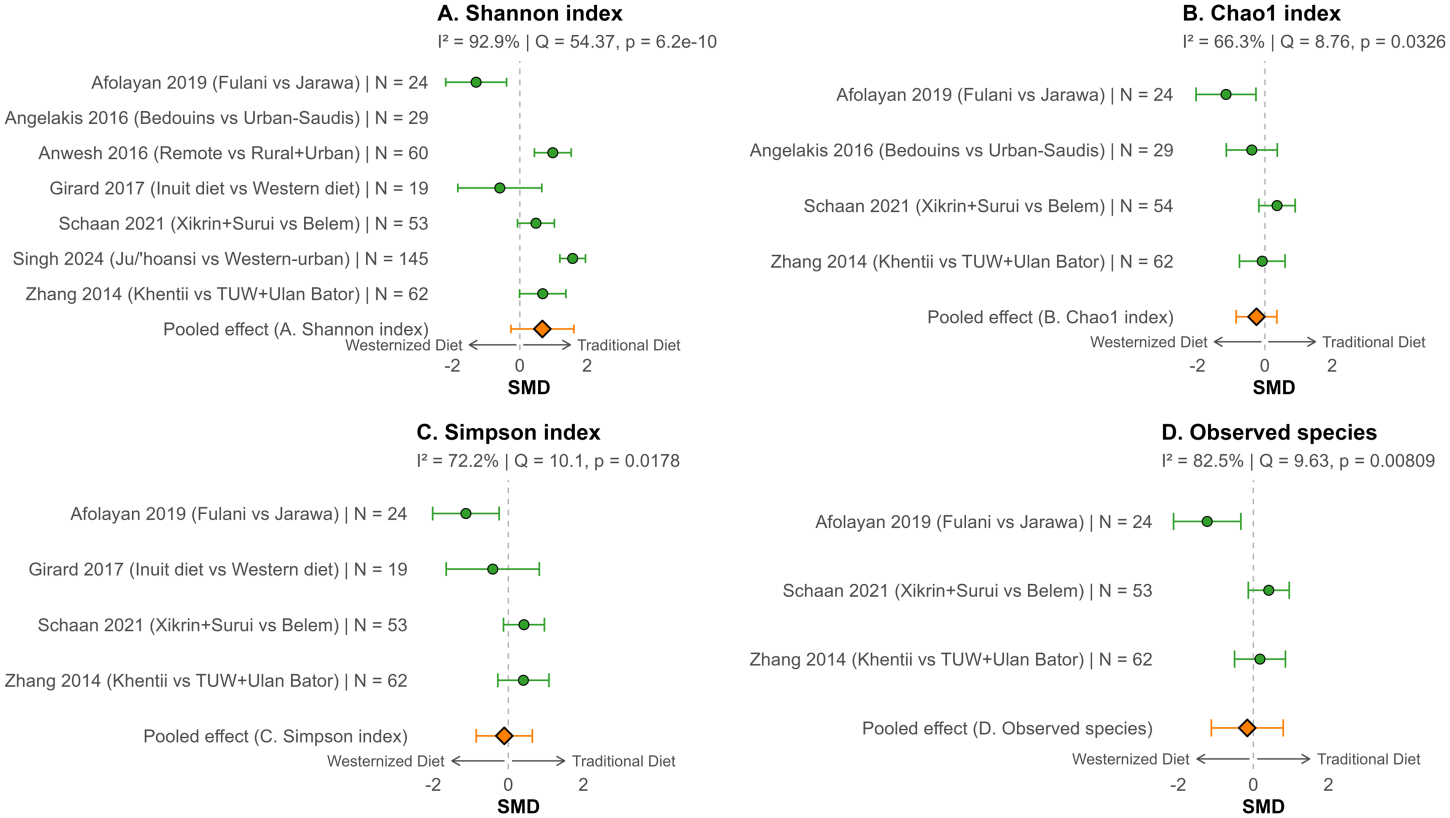
Gut microbiota diversity differences associated with dietary westernization in Indigenous populations: meta-analysis. Forest plots showing SMD in alpha-diversity indices—**(A)** Shannon, **(B)** Chao1, **(C)** Simpson, **(D)** Observed species—between Indigenous adults adhering to traditional versus westernized dietary patterns. Positive SMD values indicate higher diversity under traditional diets. Pooled estimates (orange diamonds) and study weights are shown. I^2^ values represent the percentage of total variation across studies due to heterogeneity rather than chance, with higher values (>75%) indicating considerable heterogeneity. SMD, standardized mean difference; I^2^, heterogeneity; Q, Cochran’s Q; N, sample size.

The meta-analysis revealed that gut microbiota diversity is generally higher in individuals adhering to traditional diets, although the strength and consistency of this association varied across studies and diversity metrics. For the Shannon index (Figure 4A), the pooled SMD was 0.67 (95% CI: −0.26 to 1.60) in favor of traditional diets, with very high heterogeneity (I^2^ = 92.9%, Q = 54.37, *p* < 0.001). The Chao1 index (Figure 4B) yielded a pooled SMD of −0.25 (95% CI: −0.85 to 0.36), indicating no significant difference between diet groups and moderate heterogeneity (I^2^ = 66.3%, Q = 8.76, *p* = 0.0326). The Simpson index (Figure 4C) showed a small, non-significant pooled effect (SMD = −0.11, 95% CI: −0.85 to 0.64), with high heterogeneity across studies (I^2^ = 72.2%, Q = 10.1, *p* = 0.0178). Observed species richness (Figure 4D) also showed no clear difference between groups (pooled SMD = −0.16, 95% CI: −1.12 to 0.80), with considerable heterogeneity (I^2^ = 82.5%, Q = 9.63, *p* < 0.001). Two studies could not be included in the meta-analysis because the alpha-diversity measures they used differed from those employed in the other studies. Yeo (60) used Pielou’s evenness index, while Alencar (59) assessed alpha-diversity based on the quantity of identified bacterial genera, a measure that can be considered a proxy for alpha-diversity. In Yeo’s study (60), the authors reported significantly higher alpha-diversity among Jehai hunter-gatherers compared to the most urbanized group (Jehai: 0.779 ± 0.021 vs. Temuan: 0.743 ± 0.049; *p* < 0.001). In Alencar’s study (59), no numerical alpha-diversity values were presented, and alpha-diversity could not be extracted from boxplots either. The authors qualitatively noted that Yanomami had 1.2 to 2 times greater bacterial diversity than individuals from Manaus across all age groups, although this difference was not statistically significant (*p* = 0.3972; t = 0.9115).

**Figure 5:**
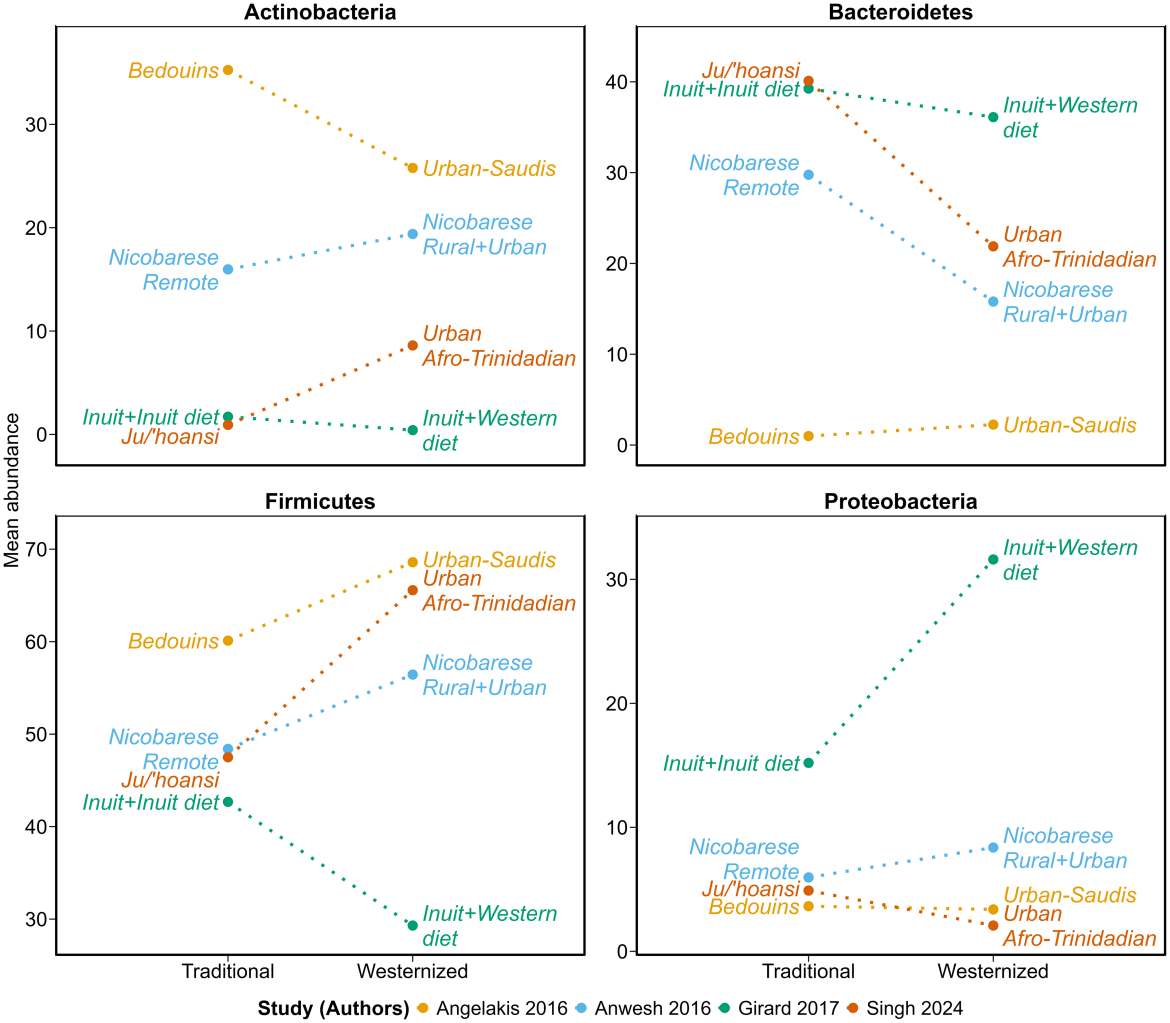
Phylum-level differences associated with dietary westernization in Indigenous populations. Slopegraphs showing the relative mean abundance of four dominant bacterial phyla—Actinobacteria, Bacteroidetes, Firmicutes, and Proteobacteria—in traditional vs. westernized groups across four independent studies (Angelakis (62); Anwesh (63); Girard (26); Singh (57)). Line colors differentiate studies. Points represent study-specific group means, and labels indicate the original group names used in each publication. Dotted lines are used to emphasize that values represent independent groups (traditional vs. westernized), and not repeated measurements within the same group (i.e., not longitudinal changes).

At the broad phylum-level, no uniform difference was observed across all studies, indicating substantial between-population variability (Figure 5). Comparisons between Bedouins and urban Saudis revealed a notable decrease in Actinobacteria (35.26 to 25.78; SDs not available) and an increase in Firmicutes (60.11 to 68.59; SDs not available), with minimal change in Bacteroidetes (1 to 2.25; SDs not available) and Proteobacteria (3.64 to 3.38; SDs not available). Among Nicobarese, the urbanized group (westernized) was associated with modest increases in Firmicutes (48.4 ± 10.58 to 56.44 ± 16.17), Actinobacteria (15.97 ± 7.31 to 19.39 ± 14.29) and Proteobacteria (5.96 ± 5.55 to 8.37 ± 11.67) levels, and a decrease in Bacteroidetes (29.76 ± 8.6 to 15.8 ± 7.78), compared to the remote group (traditional). Inuit consuming a westernized diet showed a substantial increase in Proteobacteria (15.20 ± 24.52 to 31.60 ± 48.06) and a decrease in Bacteroidetes (39.25 ± 16.02 to 36.11 ± 23.54) compared to those adhering to traditional Inuit diets, while Firmicutes (42.67 ± 17.20 to 29.31 ± 22.02) notably decreased. Ju/'hoansi and urban Afro-Trinidadians also differed markedly, with higher Actinobacteria (0.92 to 8.61; SDs not available) and Firmicutes (47.5 to 65.57; SDs not available) and lower Bacteroidetes (39.8 to 21.88; SDs not available) in the latter, and relatively stable Proteobacteria (4.9 to 2.1; SDs not available).

**Figure 6:**
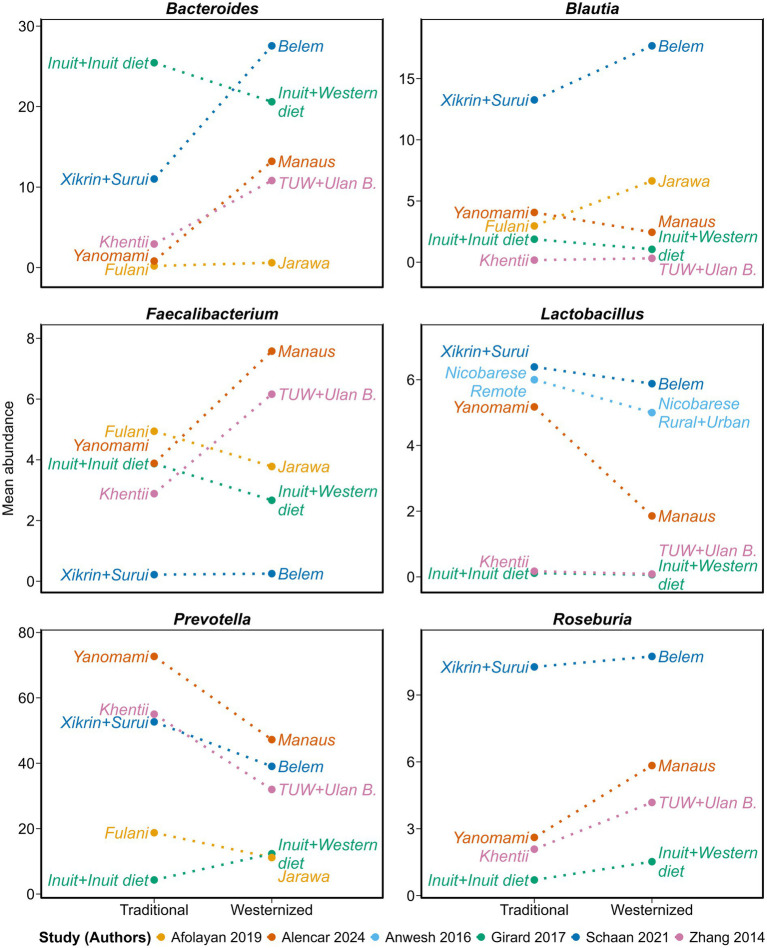
Genus-level differences associated with dietary westernization in Indigenous populations. Slopegraphs showing the relative mean abundance of six dominant bacterial genera—*Bacteroides, Blautia, Faecalibacterium, Lactobacillus, Prevotella*, and *Roseburia*—in traditional versus westernized groups across six independent studies (Afolayan (56); Alencar (59); Anwesh (63); Girard (26); Schaan (58); Zhang (61)). Line colors differentiate studies. Points represent study-specific group means, and labels indicate the original group names used in each publication. Dotted lines are used to emphasize that values represent independent groups (traditional vs. westernized), and not repeated measurements within the same group (i.e., not longitudinal changes).

In contrast to the heterogeneous shifts observed at the phylum-level (Figure 5), the genus-level responses associated with dietary westernization showed more consistent trends across populations and studies (Figure 6). Notably, *Prevotella*, a fiber-fermenting genus (65, 66), was significantly reduced in westernized groups, while *Bacteroides*, associated with animal fat and protein consumption (67), was significantly enriched. These two genera exhibited a mirror-image pattern, with *Prevotella* largely replaced by *Bacteroides* in westernized gut ecosystems, except for Inuit which exhibited the opposite pattern. Changes in other genera were subtler but still largely directional. *Faecalibacterium* and *Roseburia*, two fiber-dependent, butyrate-producing genera (68), tended to be more abundant in westernized groups, particularly in Manaus (59) and Ulan Bator (61), though their levels were lower in Jarawa and Inuit adhering to westernized dietary patterns. In contrast, *Lactobacillus* was more abundant in all traditional populations compared to their westernized counterparts, but its relative abundance was markedly lower in some groups, notably Manaus and Nicobarese (59). Overall, while the absolute abundances varied across studies, the direction of change for most genera was highly consistent, reinforcing the notion that dietary westernization exerts a predictable influence on specific components of the gut microbiota. These genus-level trends complement and extend the more variable phylum-level responses, and offer a clearer signature of microbiota restructuring in the context of traditional-to-Western dietary transitions. A limited meta-analysis based on the small subset of studies reporting genus-level data is presented in Supplementary Figure 4.

## 4 Discussion

Two decades since initial investigations into differences in gut microbiota composition between Indigenous and industrialized populations, research increasingly focuses on how dietary transitions from traditional to westernized patterns affect microbiota diversity and composition. This systematic review and meta-analysis synthesizes observational studies comparing gut microbiota diversity and composition between Indigenous populations adhering to traditional versus westernized diets, quantifying associated microbiota differences and exploring sources of variability across dietary patterns and ecological contexts.

### 4.1 Dietary westernization and microbial diversity

Although the difference was not statistically significant, our meta-analysis indicates that gut microbiota diversity was higher in individuals adhering to traditional diets, consistent with earlier observations that non-westernized groups harbor richer gut ecosystems (20, 69–71). This loss of diversity upon westernization is concerning as lower microbiota diversity has been linked to metabolic and immune dysregulation in industrialized societies (21–23). Although the pooled estimate for the Shannon index favored traditional diets, the confidence interval crossed zero, so no firm conclusion can be drawn. Still, the directionality of the effect is consistent with the idea that traditional dietary patterns promote richer microbial ecosystems. This effect was not consistent across diversity metrics: Chao1 (rare-species weighted), Simpson (evenness-weighted), and observed species richness showed no clear differences between dietary groups. These discrepancies suggest that westernization may alter the balance of mid- and low-abundance microbes (as captured by Shannon), without consistently affecting rare taxa (Chao1), total richness (observed species), or dominant species (Simpson) (72, 73). However, variability in the subset of studies available for each metric complicates direct comparisons across indices.

The lack of statistical significance observed in our analysis appears to be largely driven by the Afolayan study (56) on Fulani pastoralists, whose unusually low diversity contrasts with other traditional populations and attenuates the overall effect size. Notably, when the Afolayan study (56) was excluded, Shannon diversity became significantly higher in traditional groups (Supplementary Figure 5), supporting the idea that their data strongly influenced the overall effect, while it remained non-significant when omitting all the other studies, one at a time (Supplementary Figure 6). The authors stated that this unexpected result may stem from the Fulani’s relatively narrow, dairy-centric (11) diet, compared to their more urbanized Jarawa neighbors’ diverse diet, that include legumes, fermented grains, root vegetables, and some processed foods. Additionally, Afolayan et al. (56) reported that, compared to the Jarawa, the Fulani had a significantly higher abundance of microorganisms with predicted pathogenic potential, and notably a higher representation of the *Vibrio cholerae* pathogenicity pathway. Cholera (74) and overall pathogen exposure can cause diarrheic episodes—more prevalent among the Fulani than other Nigerian ethnic groups (75)—which likely contribute to their reduced gut microbial diversity. The high heterogeneity observed across all four metrics (I^2^ > 60%) suggests that some populations retain evenness or rare taxa despite the dietary westernization. This heterogeneity persisted even after re-running the meta-analysis without the Afolayan study (56) (Supplementary Figure 5). Methodological factors (e.g., sequencing depth, bioinformatic pipelines) and sample sizes probably also contribute to this heterogeneity.

### 4.2 Dietary westernization and microbial composition

Importantly, this non-significant tendency toward lower diversity in westernized groups was accompanied by compositional differences. Changes at both the phylum and genus-levels did not follow a single pattern but varied by population. For example, the gut microbiota of rural Bedouins (traditional diet) was dominated by Actinobacteria and Firmicutes, whereas urban Saudis showed more Proteobacteria (and a slight enrichment of Bacteroidetes). Similarly, more “westernized” Nicobarese had higher Actinobacteria and Firmicutes and lower Bacteroidetes than their traditional counterparts. Inuit adopting market foods showed especially dramatic differences: Proteobacteria bloomed while Bacteroidetes (and even Firmicutes) declined. In like manner, westernized Afro-Trinidadians carried more Actinobacteria and Firmicutes and fewer Bacteroidetes than Ju/‘hoansi hunter-gatherers. Urban Saudis, westernized Nicobarese, and westernized Afro-Trinidadians all exhibited a higher Firmicutes/Bacteroidetes ratio compared to their traditional counterparts, a pattern previously proposed as a microbial signature of dietary westernization and commonly associated with metabolic disorders and chronic inflammation (76, 77).

At the genus-level changes were remarkably consistent across studies, even more so when removing the Afolayan study (Supplementary Figure 7). *Prevotella*, a genus specializing in fiber and complex carbohydrate fermentation (65, 66), was dramatically depleted in westernized populations, whereas *Bacteroides*, a genus associated with animal fat and protein consumption (67), was enriched (see Supplementary Figure 8 for urbanization gradient). This *Prevotella*–*Bacteroides* tradeoff mirrors the classic enterotype division seen in other human cohorts: fiber-rich, high-carbohydrate diets favor *Prevotella*, whereas westernized fat- and protein-rich diets dietary patterns tend to promote *Bacteroides*. This opposing shift between *Prevotella* and *Bacteroides* constitutes a consistent microbial signature of dietary westernization. In a study tracking Southeast Asian individuals before and after their migration to the United States, adoption of American dietary habits was associated with a rapid displacement of *Prevotella* strains by *Bacteroides* strains, with significant changes detected within months of arrival (69). *Prevotella* is commonly associated with non-industrialized populations consuming fiber-rich diets, including children from Burkina Faso (78), Hadza hunter-gatherers (27), Indian vegetarians and BaAka hunter-gatherers (65), rural South Africans (24), as well as rural populations from the Amazonas of Venezuela and Malawi (79), whereas *Bacteroides* predominates in Western populations with higher intake of animal-based foods (65, 67, 69, 79). *Faecalibacterium* and *Roseburia*, two major butyrate-producing, fiber-dependent Firmicutes (68), were higher in most westernized groups. Although unexpected given the reduced fiber intake typical of westernized diets, this may reflect selective enrichment from specific fermentable fibers (80), or microbial cross-feeding interactions (81), where other bacteria degrade fibers into substrates utilized by *Faecalibacterium* and *Roseburia*, indirectly supporting their growth. As many gut-associated *Lactobacillus* species are transient and require continual reintroduction—typically via fermented foods or probiotics—their higher abundance in traditional groups may reflect greater inadvertent intake of live microbes from fermenting plant matter or even from soil on unwashed foods, in contrast to the largely sterilized westernized foods.

Overall, beyond the consistency observed in some specific genera, the high heterogeneities observed with regards to diversity metrics and dominant phyla underscore that the gut microbiota of Indigenous populations transitioning toward more westernized dietary patterns responds in population- and context-specific ways. For instance, Inuit subsist on an inherently low-fiber diet, so introducing Western foods (often still high in fat and low in fiber) may not deplete *Prevotella* as it would in groups whose traditional diet contains a lot of plants – instead we saw a marked Proteobacteria expansion, which is often a red flag for gut inflammation and dysbiosis (82). Interestingly, a higher Firmicutes/Bacteroidetes ratio and a lower *Prevotella*/*Bacteroides* ratio were observed in all westernized groups compared to their traditional counterparts, except in the Inuit. Although methodological differences across studies likely contribute to this variability (see Limitations), they are unlikely to fully explain it, as comparable results can be obtained across analysis platforms when applied to the same dataset (83).

### 4.3 Traditional diets and transition patterns

Rather, this heterogeneity presumably reflects differences in both pre-transition microbiota of the groups and overall exposome change. Two major factors to consider are the traditional diet from which the populations are shifting, as well as the way the dietary transition is unfolding (e.g., duration, magnitude, implementation) across groups.

**Figure 7:**
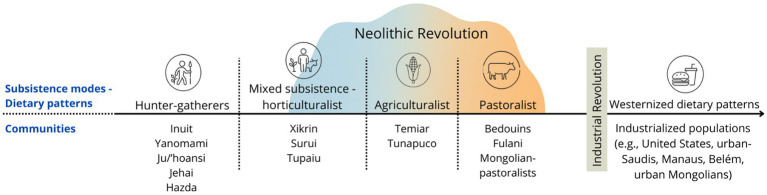
Spectrum of traditional subsistence modes/dietary patterns. Simplified view of dietary diversity prior to the Industrial Revolution. The irregular, overlapping shape representing the “Neolithic Revolution” illustrates the non-linear, heterogeneous trajectories of plant and animal domestication across populations. Subsistence categories (e.g., hunter-gatherers, pastoralists) are not rigid or mutually exclusive, as many groups occupy intermediate or mixed niches. Moreover, dietary transitions do not follow a single linear axis from foraging to westernized diets. For instance, some animal-based pastoralist diets (e.g., Bedouins, Mongols) more closely resemble those of purely animal-based hunter-gatherers (e.g., Inuit) than plant-based agriculturalist diets, despite their closer position on the spectrum. This continuum underscores that “traditional diet” is not a singular entity but rather encompasses a broad spectrum of ecological and cultural adaptations that preceded the westernized dietary shifts following the Industrial Revolution.

Importantly, while humans have continually modified their niche throughout evolution and recent history, two major dietary shifts are widely recognized: the Neolithic Revolution, which marked the transition from hunting and gathering to farming, and the Industrial Revolution. Both led to significant changes in dietary patterns and account for much of the inter-group variability observed today (Figure 7). Hence, to understand both the high diversity of traditional diets that exist across Indigenous populations, and the varying transition patterns these populations experience, we need a broad view of the evolution of the relationship between *Homo sapiens* and its diet throughout its evolutionary trajectory.

Hominins’ evolution is intrinsically linked to dietary practices (e.g., hunting, domestication of fire, food processing techniques) (84). By the time *Homo sapiens* emerged, the species had become uniquely adapted to learn, innovate, and transmit knowledge—particularly in relation to food, facilitating the construction of new dietary niches across diverse environments and thus the global dispersal of the species (85). The transition to farming, which varied extensively across regions and over time (86, 87), accelerated the diversification of human dietary niches and foodways far beyond the genetic differences observed between populations (88). Over time, hunter-gatherer groups were progressively displaced from fertile agricultural lands into less hospitable environments, such as Arctic tundras (e.g., Inuit), dense tropical forests (e.g., Jehai, Xikrin, Suruí), or semi-arid grasslands (e.g., Ju/'hoansi) (8, 32, 46). Given this historical context, the definition of "traditional diets" is inherently heterogeneous, spanning a broad spectrum—from purely hunter-gatherer diets without agricultural influences (e.g., Inuit, Hadza, Jehai), through mixed subsistence strategies combining hunting, gathering, and horticulture (e.g., Melanesian Meriam, Xikrin, Tsimane), to predominantly agricultural-based traditional diets (e.g., Temiar).

Scholars typically demarcate traditional dietary patterns based on whether they predate dietary Westernization induced by the Industrial Revolution (Figure 7). However, because the Neolithic Revolution had already variably transformed human dietary niches and consequently their gut microbiota, it remains unclear whether all groups were equally adapted to their "traditional" diets and the microbial ecosystems these diets foster (89). The Industrial Revolution marked a second major dietary upheaval, and the varying stages of dietary transition across populations significantly contribute to the heterogeneity of our results. Indigenous peoples worldwide exhibit highly diverse dietary transitions, driven by complex interactions between structural and cultural factors. The interaction of geography, politics, economics, ecology, and culture produces different trajectories of transition. Communities that have maintained greater isolation, autonomy, and knowledge of traditional foodways (often by choice or circumstance) tend to show slower transitions and better health, whereas those subjected to aggressive marginalization, globalization, and ecological stress have seen faster transitions.

### 4.4 Limitations, methodological considerations, and strengths

A key challenge inherent to observational studies, including those on the gut microbiota, is the difficulty of disentangling dietary effects from other aspects of urbanization, as most included studies did not explicitly control for, or measure variables such as access to clean water, sanitation, healthcare, or exposure to environmental pollutants. Therefore, our results may also reflect the influence of these unmeasured factors, complicating attribution to dietary patterns alone. This concern is further supported by recent multidimensional analyses showing that dietary shifts and urbanization represent distinct axes of lifestyle variation, with urbanicity (e.g., household construction materials, access to electricity, sewage infrastructure) and material wealth (e.g., ownership of televisions, mobile phones) explaining more variance in health outcomes than dietary variables alone (90). Although their study focuses on cardiometabolic outcomes rather than gut microbiota, the same reasoning may apply: if factors such as infrastructure, sanitation, pollution exposure, and healthcare access covary with diet but are not measured, attributing microbiota variation solely to dietary patterns becomes problematic.

We did not exclude studies a priori based on medication use. We recognize that some medications can affect the gut microbiota. However, this was usually not an exclusion criterion in included studies, and individual‑level data were generally unavailable to categorize participants accordingly. For each study, we extracted whether recent antibiotics and/or microbiota‑modulating medications (e.g., proton‑pump inhibitors) were exclusion criteria or reported at baseline. We treated medication/antibiotic exposure as a potential confounder in the ROBINS‑E “confounding” domain and flagged unclear or unreported exposure; this decision is reflected in our risk‑of‑bias narrative and figures. We acknowledge that medication use can still represent a confounder of our estimates of microbiota composition.

Methodological heterogeneity across studies constitutes another limitation. Differences in sequencing techniques (16S rRNA vs. shotgun metagenomics), targeted gene regions (e.g., V3–V4 vs. V4 only), sequencing depth, covariate adjustment (e.g., age, antibiotics, nutrition), bioinformatics pipelines, and varying sample sizes (12–80 participants) all potentially influence microbiota profiling, complicating comparisons. Moreover, small sample sizes within exposure and comparator groups in several studies further limited statistical power and the reliability of the observed differences. For example, in Girard et al. (26), only 16 participants were included in the traditional diet group and just 3 in the westernized group, substantially increasing the risk of random variation and sampling bias. In such cases, limited group sizes can skew diversity and abundance estimates due to outlier influence, thereby undermining the robustness and generalizability of the findings.

An additional limitation is the small number of studies included, especially for taxon-specific analyses, which precluded any formal meta-analysis. Moreover, phylum- and genus-level analyses were based on distinct subsets of studies, limiting the generalizability and coherence of taxonomic interpretations. Consequently, these results should be interpreted as exploratory patterns requiring confirmation in larger datasets. One major strength of our approach is that, unlike previous studies comparing highly distinct groups (e.g., Hadza vs. urban Americans), we included only studies with exposure and comparator groups from geographically proximate or genetically similar populations, thereby reducing confounding by genetic background and geography. This design also ensured methodological consistency in microbiota assessment, as DNA extraction, sequencing, and analysis pipelines were standardized within each study. Finally, the overall methodological quality of the included studies also represents a limitation. Three studies were rated at high risk of bias, primarily due to inadequate adjustment for confounding factors. The remaining six studies had moderate risk of bias, largely due to small sample sizes, non-random participant selection, and incomplete control of potential confounders.

## 5 Conclusion

Overall, our results indicated that gut microbial diversity was lower in individuals adhering to westernized diets compared to those following traditional diets; however, this difference was not statistically significant, and considerable heterogeneity across studies limits the strength of this conclusion. The heterogeneity of taxonomic differences likely reflects variation in traditional dietary baselines, transition trajectories, and ecological contexts across Indigenous populations. Despite this variability, some compositional patterns associated with westernization emerged across studies, most notably a higher Firmicutes/Bacteroidetes ratio, a lower *Prevotella*/*Bacteroides* and lower levels of *Lactobacillus*, suggesting a tendency toward convergent changes in the abundance of certain microbial taxa. Yet, it remains unclear how generalizable these patterns are. Future work linking microbiota variation to broader exposome components will be key to understanding the health consequences of industrialized lifestyles.

## Data Availability

The data analyzed in this study is subject to the following licenses/restrictions: the datasets analyzed in this study were extracted from previously published articles. While some of the data were publicly available, others were manually retrieved from figures when raw data were not accessible. The compiled dataset (including means, standard deviations, and sample sizes) is not currently included in the Supplementary material but can be provided by the authors upon request. Requests to access these datasets should be directed to camille.daunizeau.1@ulaval.ca.

## References

[1] Fuentes A. Human niche, human behaviour, human nature. Interface Focus. (2017) 7:20160136. doi: 10.1098/rsfs.2016.0136, 28839917 PMC5566805

[2] Low FM Gluckman PD Hanson MA. Niche modification, human cultural evolution and the Anthropocene. Trends Ecol Evol. (2019) 34:883–5. doi: 10.1016/j.tree.2019.07.005, 31422891

[3] Wild CP. Complementing the genome with an “Exposome”: the outstanding challenge of environmental exposure measurement in molecular epidemiology. Cancer Epidemiol Biomarkers Prev. (2005) 14:1847–50. doi: 10.1158/1055-9965.EPI-05-0456, 16103423

[4] Meetoo D. Chronic diseases: the silent global epidemic. Br J Nurs. (2008) 17:1320–5. doi: 10.12968/bjon.2008.17.21.31731, 19060813

[5] Gurven M Kaplan H Winking J Eid Rodriguez D Vasunilashorn S Kim JK . Inflammation and infection do not promote arterial aging and cardiovascular disease risk factors among lean horticulturalists. PLoS One. (2009) 4:e6590. doi: 10.1371/journal.pone.0006590, 19668697 PMC2722089

[6] Jones NB. Demography and evolutionary ecology of Hadza hunter-gatherers, Cambridge, UK: Cambridge University Press. vol. 71 (2016).

[7] Masterson EE Leonard WR Hujoel PP. Diet, atherosclerosis, and helmintic infection in Tsimane. Lancet. (2017) 390:2034–5. doi: 10.1016/S0140-6736(17)31945-1, 29115238

[8] Pontzer H Wood BM Raichlen DA. Hunter-gatherers as models in public health. Obes Rev. (2018) 19:24–35. doi: 10.1111/obr.12785, 30511505

[9] Singh M Raina S Goswami S Raj D. Are the tribal highlanders protected from hypertension? A meta-analysis on prevalence of hypertension among high altitude tribal population of India. Indian J Public Health. (2020) 64:295. doi: 10.4103/ijph.IJPH_509_19, 32985432

[10] Lieberman DE. The story of the human body: Evolution, health, and disease. New York, NY, USA: Vintage (2014). 27875612

[11] Glew RH Williams M Conn CA Cadena SM Crossey M Okolo SN . Cardiovascular disease risk factors and diet of Fulani pastoralists of northern Nigeria. Am J Clin Nutr. (2001) 74:730–6. doi: 10.1093/ajcn/74.6.730, 11722953

[12] Cani PD Bibiloni R Knauf C Waget A Neyrinck AM Delzenne NM . Changes in gut microbiota control metabolic endotoxemia-induced inflammation in high-fat diet–induced obesity and diabetes in mice. Diabetes. (2008) 57:1470–81. doi: 10.2337/db07-1403, 18305141

[13] Carbia C Bastiaanssen TFS Iannone LF García-Cabrerizo R Boscaini S Berding K . The microbiome-gut-brain axis regulates social cognition & craving in young binge drinkers. EBioMedicine. (2023) 89:104442. doi: 10.1016/j.ebiom.2023.104442, 36739238 PMC10025767

[14] Horn J Mayer DE Chen S Mayer EA. Role of diet and its effects on the gut microbiome in the pathophysiology of mental disorders. Transl Psychiatry. (2022) 12:164. doi: 10.1038/s41398-022-01922-0, 35443740 PMC9021202

[15] Rogers GB Keating DJ Young RL Wong M-L Licinio J Wesselingh S. From gut dysbiosis to altered brain function and mental illness: mechanisms and pathways. Mol Psychiatry. (2016) 21:738–48. doi: 10.1038/mp.2016.50, 27090305 PMC4879184

[16] Adolph TE Tilg H. Western diets and chronic diseases. Nat Med. (2024) 30:2133–47. doi: 10.1038/s41591-024-03165-6, 39085420

[17] García-Montero C Fraile-Martínez O Gómez-Lahoz AM Pekarek L Castellanos AJ Noguerales-Fraguas F . Nutritional components in western diet versus Mediterranean diet at the gut microbiota–immune system interplay. Implications for health and disease. Nutrients. (2021) 13:699. doi: 10.3390/nu13020699, 33671569 PMC7927055

[18] Martínez Leo EE Segura Campos MR. Effect of ultra-processed diet on gut microbiota and thus its role in neurodegenerative diseases. Nutrition. (2020) 71:110609. doi: 10.1016/j.nut.2019.110609, 31837645

[19] David LA Maurice CF Carmody RN Gootenberg DB Button JE Wolfe BE . Diet rapidly and reproducibly alters the human gut microbiome. Nature. (2014) 505:559–63. doi: 10.1038/nature12820, 24336217 PMC3957428

[20] Partula V Mondot S Torres MJ Kesse-Guyot E Deschasaux M Assmann K . Associations between usual diet and gut microbiota composition: results from the milieu intérieur cross-sectional study. Am J Clin Nutr. (2019) 109:1472–83. doi: 10.1093/ajcn/nqz029, 31051503

[21] Zinöcker M Lindseth I. The Western diet–microbiome-host interaction and its role in metabolic disease. Nutrients. (2018) 10:365. doi: 10.3390/nu10030365, 29562591 PMC5872783

[22] Newsome R Yang Y Jobin C. Western diet influences on microbiome and carcinogenesis. Semin Immunol. (2023) 67:101756. doi: 10.1016/j.smim.2023.101756, 37018910

[23] Severino A Tohumcu E Tamai L Dargenio P Porcari S Rondinella D . The microbiome-driven impact of western diet in the development of noncommunicable chronic disorders. Best Pract Res Clin Gastroenterol. (2024) 72:101923. doi: 10.1016/j.bpg.2024.101923, 39645277

[24] O’Keefe SJD Li JV Lahti L Ou J Carbonero F Mohammed K . Fat, fibre and cancer risk in African Americans and rural Africans. Nat Commun. (2015) 6:6342. doi: 10.1038/ncomms7342, 25919227 PMC4415091

[25] Dubois G Girard C Lapointe F-J Shapiro BJ. The Inuit gut microbiome is dynamic over time and shaped by traditional foods. Microbiome. (2017) 5:151. doi: 10.1186/s40168-017-0370-7, 29145891 PMC5689144

[26] Girard C Tromas N Amyot M Shapiro BJ. Gut microbiome of the Canadian Arctic Inuit. mSphere. (2017) 2:e00297-16. doi: 10.1128/mSphere.00297-16, 28070563 PMC5214747

[27] Schnorr SL Candela M Rampelli S Centanni M Consolandi C Basaglia G . Gut microbiome of the Hadza hunter-gatherers. Nat Commun. (2014) 5:3654. doi: 10.1038/ncomms4654, 24736369 PMC3996546

[28] Sprockett DD Martin M Costello EK Burns AR Holmes SP Gurven MD . Microbiota assembly, structure, and dynamics among Tsimane horticulturalists of the Bolivian Amazon. Nat Commun. (2020) 11:3772. doi: 10.1038/s41467-020-17541-6, 32728114 PMC7391733

[29] Obregon-Tito AJ Tito RY Metcalf J Sankaranarayanan K Clemente JC Ursell LK . Subsistence strategies in traditional societies distinguish gut microbiomes. Nat Commun. (2015) 6:6505. doi: 10.1038/ncomms7505, 25807110 PMC4386023

[30] Rampelli S Schnorr SL Consolandi C Turroni S Severgnini M Peano C . Metagenome sequencing of the Hadza hunter-gatherer gut microbiota. Curr Biol. (2015) 25:1682–93. doi: 10.1016/j.cub.2015.04.055, 25981789

[31] Smits SA Leach J Sonnenburg ED Gonzalez CG Lichtman JS Reid G . Seasonal cycling in the gut microbiome of the Hadza hunter-gatherers of Tanzania. Science. (2017) 357:802–6. doi: 10.1126/science.aan4834, 28839072 PMC5891123

[32] Haemamalar K Zalilah M Neng Azhanie A. Nutritional status of orang Asli (Che Wong tribe) adults in Krau wildlife reserve, Pahang. Malays J Nutr. (2010) 16:55–68. 22691853

[33] Kuhnlein HV Kubow S Soueida R. Lipid components of traditional Inuit foods and diets of Baffin Island. J Food Compos Anal. (1991) 4:227–36. doi: 10.1016/0889-1575(91)90034-4

[34] O’Dea KO. Traditional diet and food preferences of Australian aboriginal hunter-gatherers. Phil Trans R Soc Lond B. (1991) 334:233–41. 1685581 10.1098/rstb.1991.0112

[35] Dufour DL. Diet and nutritional status of Ameridians: a review of the literature. Cad Saúde Pública. (1991) 7:481–502. doi: 10.1590/S0102-311X1991000400003, 15798855

[36] Martin MA Lassek WD Gaulin SJC Evans RW Woo JG Geraghty SR . Fatty acid composition in the mature milk of Bolivian forager-horticulturalists: controlled comparisons with a US sample. Matern Child Nutr. (2012) 8:404–18. doi: 10.1111/j.1740-8709.2012.00412.x, 22624983 PMC3851016

[37] Dounias E Froment A. From foraging to farming among present-day forest hunter-gatherers: consequences on diet and health. Int Forest Rev. (2011) 13:294–304. doi: 10.1505/146554811798293818

[38] Gallegos-Riofrío CA Waters WF Carrasco A Riofrío LA Pintag M Caranqui M . Caliata: an indigenous Community in Ecuador Offers Lessons on food sovereignty and sustainable diets. Curr Dev Nutr. (2021) 5:61–73. doi: 10.1093/cdn/nzab009, 34222768 PMC8242225

[39] Cordain L Miller JB Eaton SB Mann N Holt SH Speth JD. Plant-animal subsistence ratios and macronutrient energy estimations in worldwide hunter-gatherer diets. Am J Clin Nutr. (2000) 71:682–92. doi: 10.1093/ajcn/71.3.682, 10702160

[40] Kuhnlein HV Receveur O. Dietary change and traditional food Systems of Indigenous Peoples. Annu Rev Nutr. (1996) 16:417–42. doi: 10.1146/annurev.nu.16.070196.002221, 8839933

[41] Kuhnlein HV Receveur O Soueida R Egeland GM. Arctic indigenous peoples experience the nutrition transition with changing dietary patterns and obesity. J Nutr. (2004) 134:1447–53. doi: 10.1093/jn/134.6.1447, 15173410

[42] Reyes-García V Powell B Díaz-Reviriego I Fernández-Llamazares Á Gallois S Gueze M. Dietary transitions among three contemporary hunter-gatherers across the tropics. Food Secur. (2019) 11:109–22. doi: 10.1007/s12571-018-0882-4

[43] Wallace IJ Lea AJ Lim YAL Chow SKW Sayed IBM Ngui R . Orang Asli health and lifeways project (OA HeLP): a cross-sectional cohort study protocol. BMJ Open. (2022) 12:e058660. doi: 10.1136/bmjopen-2021-058660, 36127083 PMC9490611

[44] Lee A. The transition of Australian aboriginal diet and nutritional health In: Simopoulos AP, editor. World review of nutrition and dietetics, vol. 79. Basel, Switzerland: S. Karger AG (1996). 1–52. 9111809

[45] Higgins J. Thomas J., Chandler J., Cumpston M., Li T., Page M. & Welch V. Cochrane handbook for systematic reviews of interventions version 6.4. Available online at: http://www.cochrane-handbook.org (2023). (Accessed June 7, 2024).

[46] PRISMA-P Group Moher D Shamseer L Clarke M Ghersi D Liberati A . Preferred reporting items for systematic review and meta-analysis protocols (PRISMA-P) 2015 statement. Syst Rev. (2015) 4:1. doi: 10.1186/2046-4053-4-1, 25554246 PMC4320440

[47] Cobo M Jose R UN. Subcommission on Prevention of Discrimination and Protection of Minorities. Special Rapporteur on the Problem of Discrimination against Indigenous Populations. Study of the problem of discrimination against indigenous populations. Volume 5, Conclusions, proposals and recommendations. New York, NY, USA: United Nations Digital Library (1987). 46 p.

[48] United Nations, United Nations declaration on the rights of indigenous peoples. United Nations. New York, NY, USA: UN Digital Library imprint. (2007) 1–18.

[49] Rocillo-Aquino Z Cervantes-Escoto F Leos-Rodríguez JA Cruz-Delgado D Espinoza-Ortega A. What is a traditional food? Conceptual evolution from four dimensions. J Ethn Food. (2021) 8:38. doi: 10.1186/s42779-021-00113-4

[50] Sproesser G Ruby MB Arbit N Akotia CS Alvarenga MDS Bhangaokar R . Understanding traditional and modern eating: the TEP10 framework. BMC Public Health. (2019) 19:1606. doi: 10.1186/s12889-019-7844-4, 31791293 PMC6889524

[51] Carrera-Bastos P Fontes O O’Keefe P Lindeberg Y Cordain S. The western diet and lifestyle and diseases of civilization. RRCC. (2011) 15:919. doi: 10.2147/RRCC.S16919

[52] Hozo SP Djulbegovic B Hozo I. Estimating the mean and variance from the median, range, and the size of a sample. BMC Med Res Methodol. (2005) 5:13. doi: 10.1186/1471-2288-5-13, 15840177 PMC1097734

[53] R Core Team. R: A Language and Environment for Statistical Computing. Vienna, Austria: R Foundation for Statistical Computing (2023). Available at: https://www.R-project.org/

[54] Kontopantelis E Reeves D. Performance of statistical methods for meta-analysis when true study effects are non-normally distributed: a simulation study. Stat Methods Med Res. (2012) 21:409–26. doi: 10.1177/0962280210392008, 21148194

[55] Kontopantelis E Reeves D. Performance of statistical methods for meta-analysis when true study effects are non-normally distributed: a comparison between DerSimonian–Laird and restricted maximum likelihood. Stat Methods Med Res. (2012) 21:657–9. doi: 10.1177/0962280211413451, 23171971

[56] Afolayan AO Ayeni FA Moissl-Eichinger C Gorkiewicz G Halwachs B Högenauer C. Impact of a nomadic pastoral lifestyle on the gut microbiome in the Fulani living in Nigeria. Front Microbiol. (2019) 10:2138. doi: 10.3389/fmicb.2019.02138, 31572342 PMC6753190

[57] Singh H. Wiscovitch-Russo R. Kuelbs C. Espinoza J. Appel A. E. Lyons R. J. Multiomic insights into human health: gut microbiomes of hunter-gatherer, agropastoral, and Western urban populations. bioRxiv [Preprint]. (2024).

[58] Schaan AP Sarquis D Cavalcante GC Magalhães L Sacuena ERP Costa J . The structure of Brazilian Amazonian gut microbiomes in the process of urbanisation. NPJ Biofilms Microbiomes. (2021) 7:65. doi: 10.1038/s41522-021-00237-0, 34354062 PMC8342711

[59] Alencar RM Martínez JG Machado VN Alzate JF Ortiz-Ojeda CP Matias RR . Preliminary profile of the gut microbiota from amerindians in the Brazilian amazon experiencing a process of transition to urbanization. Braz J Microbiol. (2024) 55:2345–54. doi: 10.1007/s42770-024-01413-y, 38913252 PMC11405645

[60] Yeo L-F Lee SC Palanisamy UD Khalid B Ayub Q Lim SY . The Oral, gut microbiota and Cardiometabolic health of indigenous orang Asli communities. Front Cell Infect Microbiol. (2022) 12:812345. doi: 10.3389/fcimb.2022.812345, 35531342 PMC9074829

[61] Zhang J Guo Z Lim AAQ Zheng Y Koh EY Ho D . Mongolians core gut microbiota and its correlation with seasonal dietary changes. Sci Rep. (2014) 4:5001. doi: 10.1038/srep05001 24833488 PMC4023135

[62] Angelakis E Yasir M Bachar D Azhar EI Lagier J-C Bibi F . Gut microbiome and dietary patterns in different Saudi populations and monkeys. Sci Rep. (2016) 6:32191. doi: 10.1038/srep32191, 27578328 PMC5006041

[63] Anwesh M Kumar KV Nagarajan M Chander MP Kartick C Paluru V. Elucidating the richness of bacterial groups in the gut of Nicobarese tribal community – perspective on their lifestyle transition. Anaerobe. (2016) 39:68–76. doi: 10.1016/j.anaerobe.2016.03.002, 26946360

[64] Ukhnaa T. Damba G. Protection of Minority Rights in Mongolia. Available online at: http://www.eai.or.kr (2022). (Accessed April 15, 2025).

[65] Prasoodanan PK Sharma AK Mahajan S Dhakan DB Maji A Scaria J . Western and non-western gut microbiomes reveal new roles of Prevotella in carbohydrate metabolism and mouth–gut axis. NPJ Biofilms Microbiomes. (2021) 7:77. doi: 10.1038/s41522-021-00248-x, 34620880 PMC8497558

[66] Chen T Long W Zhang C Liu S Zhao L Hamaker BR. Fiber-utilizing capacity varies in Prevotella- versus Bacteroides-dominated gut microbiota. Sci Rep. (2017) 7:2594. doi: 10.1038/s41598-017-02995-4, 28572676 PMC5453967

[67] Reddy BS Weisburger JH Wynder EL. Effects of high risk and Low risk diets for Colon carcinogenesis on fecal microflora and steroids in man. J Nutr. (1975) 105:878–84. doi: 10.1093/jn/105.7.878, 1138032

[68] Machiels K Joossens M Sabino J De Preter V Arijs I Eeckhaut V . A decrease of the butyrate-producing species *Roseburia hominis* and *Faecalibacterium prausnitzii* defines dysbiosis in patients with ulcerative colitis. Gut. (2014) 63:1275–83. doi: 10.1136/gutjnl-2013-304833, 24021287

[69] Vangay P Johnson AJ Ward TL Al-Ghalith GA Shields-Cutler RR Hillmann BM . US immigration westernizes the human gut microbiome. Cell. (2018) 175:962–972.e10. doi: 10.1016/j.cell.2018.10.029, 30388453 PMC6498444

[70] Gao J Guo X Wei W Li R Hu K Liu X . The Association of Fried Meat Consumption with the gut microbiota and fecal metabolites and its impact on glucose homoeostasis, intestinal endotoxin levels, and systemic inflammation: a randomized controlled-feeding trial. Diabetes Care. (2021) 44:1970–9. doi: 10.2337/dc21-0099, 34253560

[71] Turnbaugh PJ Bäckhed F Fulton L Gordon JI. Diet-induced obesity is linked to marked but reversible alterations in the mouse distal gut microbiome. Cell Host Microbe. (2008) 3:213–23. doi: 10.1016/j.chom.2008.02.015, 18407065 PMC3687783

[72] Kers JG Saccenti E. The power of microbiome studies: some considerations on which alpha and Beta metrics to use and how to report results. Front Microbiol. (2022) 12:796025. doi: 10.3389/fmicb.2021.796025, 35310396 PMC8928147

[73] Morris EK Caruso T Buscot F Fischer M Hancock C Maier TS . Choosing and using diversity indices: insights for ecological applications from the German biodiversity Exploratories. Ecol Evol. (2014) 4:3514–24. doi: 10.1002/ece3.1155, 25478144 PMC4224527

[74] Cho JY Liu R Macbeth JC Hsiao A. The Interface of *vibrio cholerae* and the gut microbiome. Gut Microbes. (2021) 13:1937015. doi: 10.1080/19490976.2021.1937015, 34180341 PMC8244777

[75] Fayehun O Omololu O. Prevalence and treatment of childhood diarrhea among Nigerian ethnic groups. Niger J Sociol Anthropol. (2009) 7:160. doi: 10.36108/NJSA/9002/70(0160)

[76] Nakayama J Yamamoto A Palermo-Conde LA Higashi K Sonomoto K Tan J . Impact of westernized diet on gut microbiota in children on Leyte Island. Front Microbiol. (2017) 8:197. doi: 10.3389/fmicb.2017.00197, 28261164 PMC5306386

[77] Petakh P Oksenych V Kamyshnyi A. The F/B ratio as a biomarker for inflammation in COVID-19 and T2D: impact of metformin. Biomed Pharmacother. (2023) 163:114892. doi: 10.1016/j.biopha.2023.114892, 37196542 PMC10183625

[78] De Filippo C Cavalieri D Di Paola M Ramazzotti M Poullet JB Massart S . Impact of diet in shaping gut microbiota revealed by a comparative study in children from Europe and rural Africa. Proc Natl Acad Sci USA. (2010) 107:14691–6. doi: 10.1073/pnas.1005963107, 20679230 PMC2930426

[79] Yatsunenko T Rey FE Manary MJ Trehan I Dominguez-Bello MG Contreras M . Human gut microbiome viewed across age and geography. Nature. (2012) 486:222–7. doi: 10.1038/nature11053, 22699611 PMC3376388

[80] La Rosa SL Leth ML Michalak L Hansen ME Pudlo NA Glowacki R . The human gut Firmicute *Roseburia intestinalis* is a primary degrader of dietary β-mannans. Nat Commun. (2019) 10:905. doi: 10.1038/s41467-019-08812-y, 30796211 PMC6385246

[81] Culp EJ Goodman AL. Cross-feeding in the gut microbiome: ecology and mechanisms. Cell Host Microbe. (2023) 31:485–99. doi: 10.1016/j.chom.2023.03.016, 37054671 PMC10125260

[82] Shin N-R Whon TW Bae J-W. Proteobacteria: microbial signature of dysbiosis in gut microbiota. Trends Biotechnol. (2015) 33:496–503. doi: 10.1016/j.tibtech.2015.06.011, 26210164

[83] Lehr K Oosterlinck B Then CK Gemmell MR Gedgaudas R Bornschein J . Comparison of different microbiome analysis pipelines to validate their reproducibility of gastric mucosal microbiome composition. mSystems. (2025) 10:e0135824. doi: 10.1128/msystems.01358-24, 39873520 PMC11834405

[84] Aiello LC Wheeler P. The expensive-tissue hypothesis: the brain and the digestive system in human and primate evolution. Curr Anthropol. (1995) 36:199–221. doi: 10.1086/204350

[85] Harris M Ross EB. Food and evolution: Toward a theory of human food habits. Philadelphia, PA, USA: Temple University Press (1987).

[86] Henderson DA Baggaley AW Shukurov A Boys RJ Sarson GR Golightly A. Regional variations in the European Neolithic dispersal: the role of the coastlines. Antiquity. (2014) 88:1291–302. doi: 10.1017/S0003598X00115467

[87] Münster A Knipper C Oelze VM Nicklisch N Stecher M Schlenker B . 4000 years of human dietary evolution in Central Germany, from the first farmers to the first elites. PLoS One. (2018) 13:e0194862. doi: 10.1371/journal.pone.0194862, 29584767 PMC5870995

[88] Wells JCK Stock JT. Life history transitions at the origins of agriculture: a model for understanding how niche construction impacts human growth, demography and health. Front Endocrinol. (2020) 11:325. doi: 10.3389/fendo.2020.00325, 32508752 PMC7253633

[89] Franck M De Toro-Martín J Vohl M-C. Eco-evolutionary dynamics of the human-gut microbiota symbiosis in a changing nutritional environment. Evol Biol. (2022) 49:255–64. doi: 10.1007/s11692-022-09569-x

[90] Watowich M. M. Arner A. M. Wang S. John E. Kahumbu J. C. Kinyua P. . The built environment is more predictive of cardiometabolic health than other aspects of lifestyle in two rapidly transitioning indigenous populations. medRxiv [Preprint]. (2024).

