# Supplementary material for: The gut microbiota of Indigenous populations in the context of dietary westernization: a systematic review and meta-analysis
Source: Front Nutr. 2025 Oct 22;12:1652598. doi: 10.3389/fnut.2025.1652598 (PMC12612841; doi:10.3389/fnut.2025.1652598)
Supplement: Supplementary file 1 [file Data_Sheet_1.docx]

**Supplementary materials**

**Supplementary Table 1. Search strategy for MEDLINE/Ovid performed on March 18, 2024 and February 25, 2025.**

| **Search line** | **Query** |
| --- | --- |
| *Indigenous or remote populations* | |
| 1 | (((autocht* or indigenous or native* or Ethnic) adj4 (people* or communit* or person* or population or men or man or women or woman or adult* or child* or infant* or elder* or reserv* or individu* or group* or band* or american* or canadian* or russ* or siber*)) or (Aborigin* or Aleut* or Amerind* or Eskimo* or Indian* or Inuit* or Innuit* or Inuk or Inuktitut-speaker* or Inupiat* or "First Nation*" or "First People*" or Kalaallit* or Metis or tribe or tribes or tribal)).ti,ab,kf. |
| 2 | (((isolated or remote) adj2 (area or areas or population* or communit* or goup* or reserv* or island* or land*)) or rural or urban* or non-industrial*).ti,ab,kf. |
| 3 | (Abenaki* or Pima or Pimas or Navajo* or Inuit* or Innuit* or Cherokee* or Shawnee* or Lakota or Coushatta or Tlingit* or Arapaho* or Assiniboine or Beothuk or Blackfeet or Blackfoot or Cabazon* or Caddo* or Chickasaw or Chippewa or Chitimacha* or Choctaw* or Cocopah* or "Coeur D'alene" or Comanche* or Muscogee* or Duwamish or Elwha* or Flathead* or Goshute* or Ho-Chunk* or Hopi or Hopis or Hoopa* or S'klallam or Jatibonicu or Jumano* or Kalapuya* or Kiowa* or Kootenai* or Lemhi* or Shoshone* or Makah* or Pequot* or Mechoopda* or Metis or Menominee* or Miccosukee* or MicMac or Mi'kmaq or Mohegan* or Muscogee* or Navajo* or "Nez Perce" or "Nez Pierce" or Oneida* or Osage* or Passamaquoddy or Pawnee* or "Pend D'oreille" or Pomo or Pomos or Potawatomi* or Pueblo* or Quinault* or Salish or Saponi or Saponis or Seminole* or Shawnee* or Shoshone* or Siletz or Nakota or S'klallam or Suquamish or Taino or Tainos or Tohono or "O'Odham" or Tunica-Biloxi or Tunicas or Umatilla* or Umpqua* or Waccamaw* or Wampanoag* or Washoe* or Wiyot* or Yakama* or Ute or Utes or Apache* or Cheyenne* or Spokane or Tuscarora or Sioux or Wendat* or Huron* or Mohawk* or "AI/AN" or Apache or Cree).ti,ab,kf. |
| 4 | ("A' ani" or Absaroka or Haaninin or Atsina or "Gros Ventre" or Acopsel or Tlacopsel or Lacopsel or Ahtna or Ahtena or Akenitsi or Occaneechi or Akokisa or Horcoquisa or Orcoquizas or Aleut or Unangax or Unangan or Alibamu or "Alabama Alsea" or Alutiiq or Sugpiag or "Pacific Yupik" or Amahami or Awaxawi or Androscoggin or Arosaguntacook or Ameriscoggin or Anishinaabeg or Chippewa or Anihsinape or Saulteaux or Apalachee or Aranama or "Texan Coahuilteca" or Tamique or Arikara or Sahnish or Arickaree or Adakadaho or Assiniboine or Hohe or Nakota or Nakoda or Nakona or "Atsa' Kudok-wa" or Awatixa or Bannock or "Snake Indian*" or Bidai or Quasmigdo or Biloxi or Blackfoot or Niitsitapi or Sikasikaitsitapi or Cahto or Kaipomo or Cahuilla or Ivilyuqaletem or Ivilyuat or Catawba or Inna or Iswa or Chemehuevi or Chickasaw or "Chilula Chimakum" or Aqokulo or Chimariko or Chiricahua or Tsokanende or Chitimacha or Chetimachan or Sitimacha or Chowanoke or Roanoke or Chumash or Ciboney or "Taino Ciwat" or Clatsop or Coos or Coosa or Uchis or Chiaha or Coste or Talisi or Coquille or Kokwell or Coso or Cowlitz or Taitnapam or "Crow Nation" or "Cui Ui Ticutta" or Cupeno or Kuupangaxwichem or Cupa or "Cup' ig" or Nunivak or "Dakota Oyate" or Lakota or Nakota or Santee or Teton or Sioux or Deadose or "Deg Xina" or "Deg Xit' an" or Kaiyuhkhotana or "Deg Hit' an" or "Dena' ina" or Tanaina or "Dichinanek' Hwt' ana" or "Upper Kuskokwim Athabascan*" or Kolchan or Goltsan or "Tundra Kolosh" or "Do lkabya" or Duwamish or Esselen or Eyak or "Gidi' tikadi" or Guwevkabaya or "Gwich' in" or Kutchin or Haida or Xaadas or Xaat or Halchidhoma or Havasupai or "Green Water People" or Hiratsa or Hiraaca or "Ho-chaaqa" or Winnebago or Holikachuk or Innoko or "Tlegon-khotana" or Hopi or "Houma-Louisiana" or Huaco or Waco or Hualapai or Hupa or Natinixwe or "Natinook-wa" or "Hwech' in" or Hankutchin or "Iroquois Confederacy" or "Hodinoso ni" or "Illinois Confedera*" or Ilinoweg or Illini or Inupiat or Inuit or Ioway or Baxoje or Jicarilla or Juaneno or Acjachemen or Jumano or Kalapuya or Clackama or Kalispel or "Pend d' Oreilles" or Qlispe or Karuk or Karok or "Chum-ne" or Katkoc or Kansa or Kanza or Kawaiisu or Nuwa or Kennebec or "Kinipekw Kittitas" or Klickitat or "Qwu' lh-hwai-pum" or "Awi-adshi" or Mahane or Wahnookt or "Koa' aga' itoka" or Keresan or Kichai or Kitsai or Keechi or "K' itaish" or Kiowa or Gaigwu or Cauigu or Kutjau or "Kwu-da" or "Tep-da" or Kitanemuk or Kittitas or Klickitat or "Qwu' lh-hwai-pum" or "Awi-adshi" or Mahane or Wahnookt or "Koa' aga' itoka" or Konkow or "Koop Ticutta" or Koyukon or Ktunaxa or Kootenai or Flathead or Kucadikadi or "Kotsa' va" or Kumeyaay or "Tipai-Ipai" or Kamia or Diegueno or Kwapa or Cocopah or Cucapa or "Xawitt kwnchawaay" or Lassik or Lenape or "Leni-Lenape" or Lipan or Luiseno or Payomkawichum or Madqwadabaya or "Desert Yavapai" or Mahican or Mohicans or Makah or Makuhadokado or Maliseet or Wolistoqiag or Manahoac or Mahock or Meipontsky or Mandan or Mattole or "Bear River" or "Tul' bush" or "Ni' ekeni" or Meherrin or Menominee or Mackinac or Mescalero or Myaamiaki or Kickapoo or Twigtwee or Missouria or Miwok or Miwuk or Moadokado or Modoc or Mohave or "Aha Makhav" or Mohawk or "Kaneng' hega" or Molala or Molale or Molele or Nyyhmy or Moosonee or "Moose Cree" or Monsonis or Multnomah or Chinook or Nabedache or Nabaydacu or Wawadishe or Nabiltse or Dakubetede or "Nacho Nyak Dun" or Tutchone or Nacono or "Na' isha" or Nanticoke or Navajo or Ndee or Nial or Niimiipu or "Nez Perce" or Watapala or Watapahlute or Nisenan or Nisqually or Nomlaki or Noamlakee or "Central Wintun" or Nongatl or Nottoway or Cheroenhaka or "Northern Cheyenne" or Ohlone or Costanoan or Omaha or "O' odham" or Pima or Papago or Osage or Otoe or Otse or "Ozav Dika" or Palus or Passamaquoddy or Pestomuhkati or Patiri or Petaros or Pastia or Patwin or "Southern Wintun" or Panis or Skidi or Pedee or Penobscot or "Petun Piipaash" or "Kokmalik' op" or Piscatawa or Doeg or Conoy or "Pit River" or Pomo or Kashaya or Ponca or Ponka or Pottawatomi or Bodewadmik or Powhatan or Puyallup or Spuyalepabs or Quapaw or Ugahxpa or Quechan or Yuma or Kwtsaan or Quileute or Salinan or Saponi or Monacan or Sapon or "Eastern Blackfoot" or Christanna or Sawawatodo or Serrano or Taaqtam or "Maarenga' yam" or Yuhaviatam or Shasta or Chasta or Sasti or Shoshone or Siletz or Sinkine or Sinkyone or "Siuslaw Umpqua" or Skitswish or "Schitsu' umash" or Snohomish or Snuqualmi or Sokoki or Missiquoi or Stillaguamish or Stoluckwamish or Suquamish or Sutaio or Swinomish or Skagit or Syilx or Okanagan or Sotaae or "Taga Ticutta" or Takelma or Dagelma or Taltushtuntede or Galice or "Tanan Gwich' in" or Taos or Taovaya or Tataviam or Alliklik or Tawakoni or Tahuacano or Tenino or Thawikila or Hathawekela or "Fort Ancient" or Tigua or Tillamook or Nehalem or Timbisha or Panamint or Timpanogos or Tlingit or "Toi Ticutta" or Tolowa or "Talawa Dini" or Tongva or Gabrieleno or Fernandeno or Tobikhar or Tonkawa or Ticanwatic or Tsikip or Appalousa or Opelousa or Tsitsistas or Tubatulabal or Tukabatchee or Tuscarora or Tomahittan or Kuskarawock or Tutelo or Tutero or Totteroy or Tutera or Yusan or Tututni or Umatilla or Umpqua or Waccamaw or Waxmaw or Wadatika or "Harney Valley Paiute" or Wailiki or Waluulapam or "Walla Walla" or Walpapi or Huipui or Wampanoag or Massasoit or Wanapum or Wappo or Washoe or Wichita or Willapa or Kwalhioqua or "Wi pukba" or "Verde Valley Yavapai" or Wintu or "Northern Wintun" or Wiyot or "Wee' at" or Weyet or Yakama or "Yamosopo Tuviwarai" or Yaqui or Yoeme or Yatasi or Yattasih or "Yavbe' " or "Yavapai" or "Ysleta del Sur" or Yojuane or Yokuts or Mariposa or Yuki or Yupighyt or Yup'ik or Yupik or Yurok or "Olekwo'l" or Zuni).ti,ab,kf. |
| 5 | ("Kalaallit Nunaat" or Nuuk or Sisimiut or Ilulissat or Qaqortoq Aasiaat or Maniitsoq or Tasiilaq or Uummannaq or Narsaq or Paamiut or Nanortalik or Upernavik or Qasigiannguit or Greenlandic or Kalaallit or Kalaallisut or Tunumiit or Inughuit or Avanersuarmiut).ti,ab,kf. |
| 6 | (Ainus or Ainu or Aleuts or Alyutors or Chukchis or Chuvans or Dolgans or Enets or Entsy or Yupik or "Yup'ik" or Yuit or Yupigyt or Chaplino or Naukan or Itelmens or Kamchadals or Kereks or "Komi" or Koryaks or Nenets or Nentsy or Nganasans or Tavgi or Sami or Veps or Yukaghirs or Chulyms or Evenks or Tungus or Evens or "Kets" or Khantys or Mansi or Vguls or Selkups or Teleuts or Nanais or Nanaitsy or Negidal or Nivikh or Oroch or orok or Taz or udege or ulch or Kumadins or Chelkans or Shorians or Soyots or Telengits or Tofalars or Tugalars or "Tufans" or "Todzhins" or Laks or Tabasarans or Turuls or Aguls or Tsakhurs or Kumyks or Nogais or "Andis" or Akhvakh or Archins or Bagvalals or Bezhta or Botlikhs or Chamalals or Godoberi or Hinukh or Hunzibs or Khwarshi or Karata or Tindis or Tsez or Abazin or Besermyan or Izhorians or Karelians or Nagaybaks or Setos or Shapsugs or Quratay).ti,ab,kf. |
| 7 | exp Indigenous Peoples/ or Rural Population/ |
| 8 | 1 or 2 or 3 or 4 or 5 or 6 or 7 |
| *Traditional food* | |
| 9 | (diet* or food* or feed* or fed or eat* or ate or aliment* or nourish* or nutri* or Regim* or intake or recipe* or Sustenance* or dish or dishes or beverage* or meal* or drink* or drank or consumption* or lifestyle* or "life style*").ti,ab,kf. |
| 10 | (nutrient* or macronutrient* or macro-nutrient* or micronutrient* or micro-nutrient* or vitamin* or mineral* or lactose or gluten* or omega-3 or omega-6 or "fatty acid*" or iron or prebiotic* or probiotic* or antioxidant*).ti,ab,kf. |
| 11 | (sugar* or seaweed* or algae or seafood* or "game animal*" or venison* or "wild berr*" or seafood* or "marine mammal*" or hunt* or fish* or herd* or forage* or cultivat* or harvest* or vegetable* or meat* or cereal* or fruit* or grain or grains or milk* or dairy).ti,ab,kf. |
| 12 | exp Diet/ or exp Feeding Behavior/ or Nutritional Status/ or exp "Food and Beverages"/ or "Diet, Food, and Nutrition"/ or Eating/ or Life Style/ |
| 13 | 9 or 10 or 11 or 12 |
| *Gut microbiota composition* | |
| 14 | ((gastrointestinal or gastro-intestinal or intestin* or colon or colonic or gut or bowel* or human or enteric or commensal or gastric* or fecal or feces or alimentary or digestiv*) adj3 (microb* or bacteri* or flora or microflora or micro-flora or microorganism* or micro-organism* or ecosystem*)).ti,ab,kf. |
| 15 | ((microb* or microorganism* or micro-organism*) adj2 (diversit* or communit*)).ti,ab,kf. |
| 16 | Gastrointestinal Microbiome/ |
| 17 | 14 or 15 or 16 |
| 18 | 8 and 13 and 17 |
| 19 | exp animals/ not humans.sh. |
| 20 | 18 not 19 |

**Supplementary details on data extraction and preparation**

For Girard’s study (1), relative abundances were computed from the raw OTU table available in the article by normalizing taxon counts to total reads per sample, yielding percentages. In Zhang’s study (2), individual-level alpha diversity data were available at five time points (January, March, June, September, and November). For each diversity metric, we computed the mean of individual values from the January time point (baseline), rather than averaging across all five measurements. In some studies (2–4), multiple comparator groups were available. To increase sample sizes, certain groups with similar characteristics were combined. In Schaan’s study (3), the Xikrin and Suruí groups, identified as the most traditional populations, were merged. In contrast, in Zhang (2) and Anwesh (4), rural and urban groups were pooled. In Zhang’s study (2), rural and urban participants originated from the suburbs and city center of Ulan-Bator, respectively, and shared common access to industrialized products and a partially urbanized or urban lifestyle. Similarly, in Anwesh’s study (4), rural participants exhibited lifestyles more comparable to urban individuals than to the remote Nicobarese group.

*
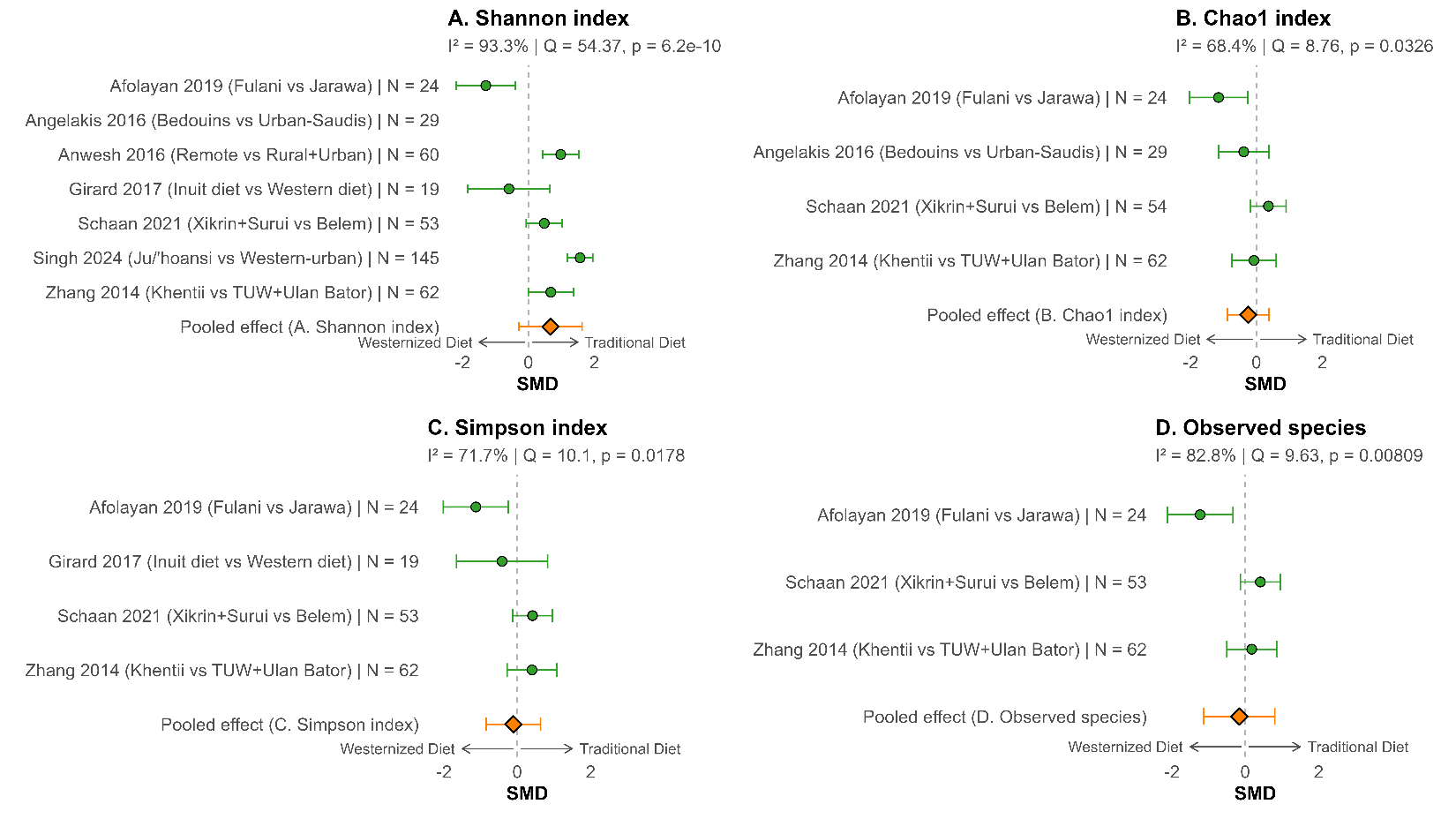
*

**Supplementary Figure 1. Gut microbiota diversity differences associated with dietary westernization in Indigenous populations: sensitivity analysis using the Sidik-Jonkman estimator.** Forest plots showing SMD in alpha-diversity indices—(A) Shannon, (B) Chao1, (C) Simpson, (D) Observed species—between Indigenous adults adhering to traditional versus more westernized dietary patterns. Positive SMD values indicate higher diversity under traditional diets. Pooled estimates (orange diamonds) and study weights are shown. I² values represent the percentage of total variation across studies due to heterogeneity rather than chance, with higher values (>75%) indicating considerable heterogeneity. Dotted lines are used to emphasize that values represent independent groups (traditional vs. westernized), and not repeated measurements within the same group (i.e., not longitudinal changes). SMD, standardized mean difference; I², heterogeneity; Q, Cochran’s Q; N, sample size.


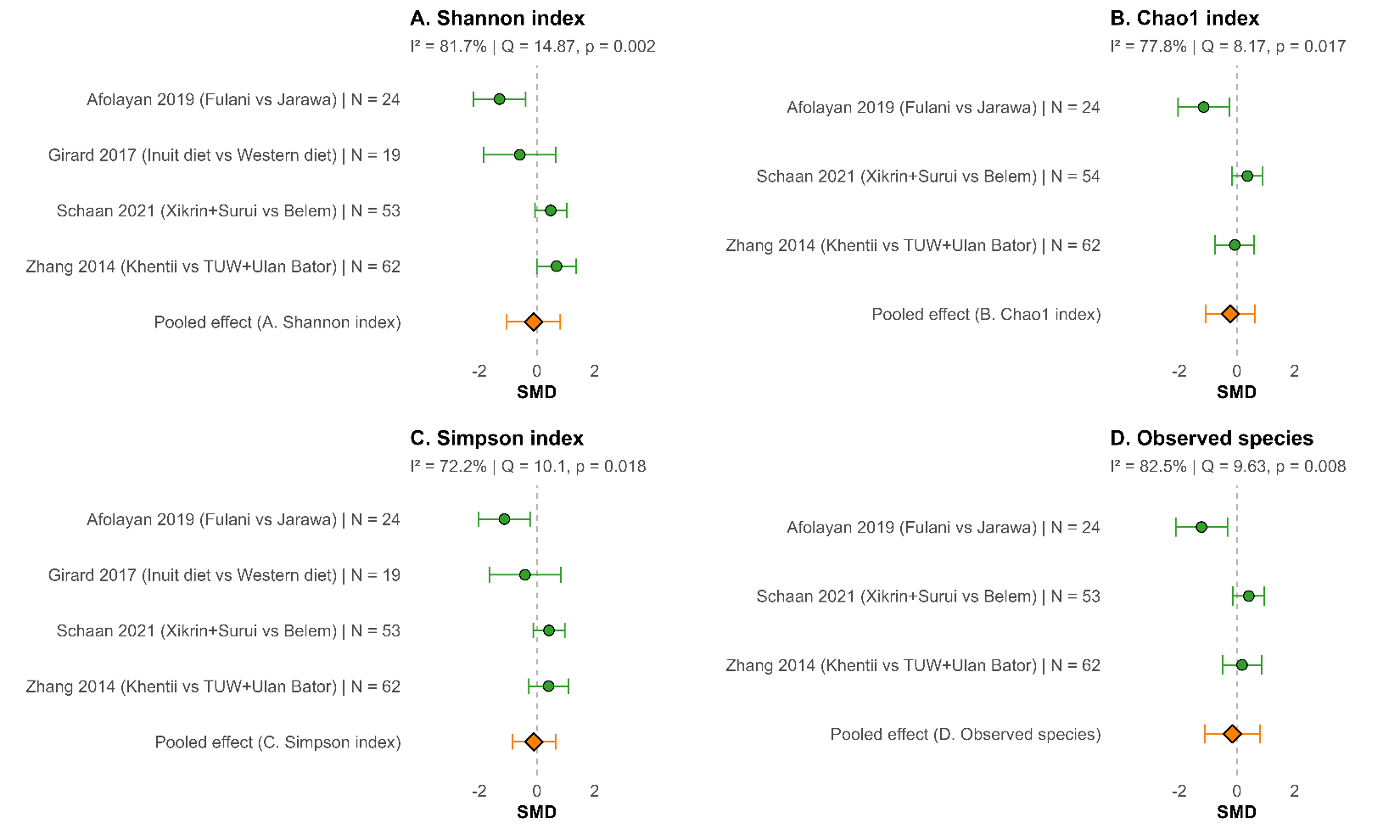


**Supplementary Figure 2.** **Gut microbiota diversity shifts associated with dietary westernization in Indigenous populations: sensitivity meta-analysis excluding older participants (>65 years).** Forest plots showing SMD in alpha-diversity indices—(A) Shannon, (B) Chao1, (C) Simpson, (D) Observed species—between Indigenous adults (16–65 years) adhering to traditional versus more westernized dietary patterns. Positive SMD values indicate higher diversity under traditional diets. Pooled estimates (orange diamonds) and study weights are shown. Each meta‑analysis used a random‑effects model with the restricted maximum likelihood (REML) estimator. I² values represent the percentage of total variation across studies due to heterogeneity rather than chance, with higher values (>75%) indicating considerable heterogeneity. Studies by Angelakis (5), Anwesh (4), and Singh (6) were excluded to remove participants older than 65 years. SMD, standardized mean difference; I², heterogeneity; Q, Cochran’s Q; N, sample size.


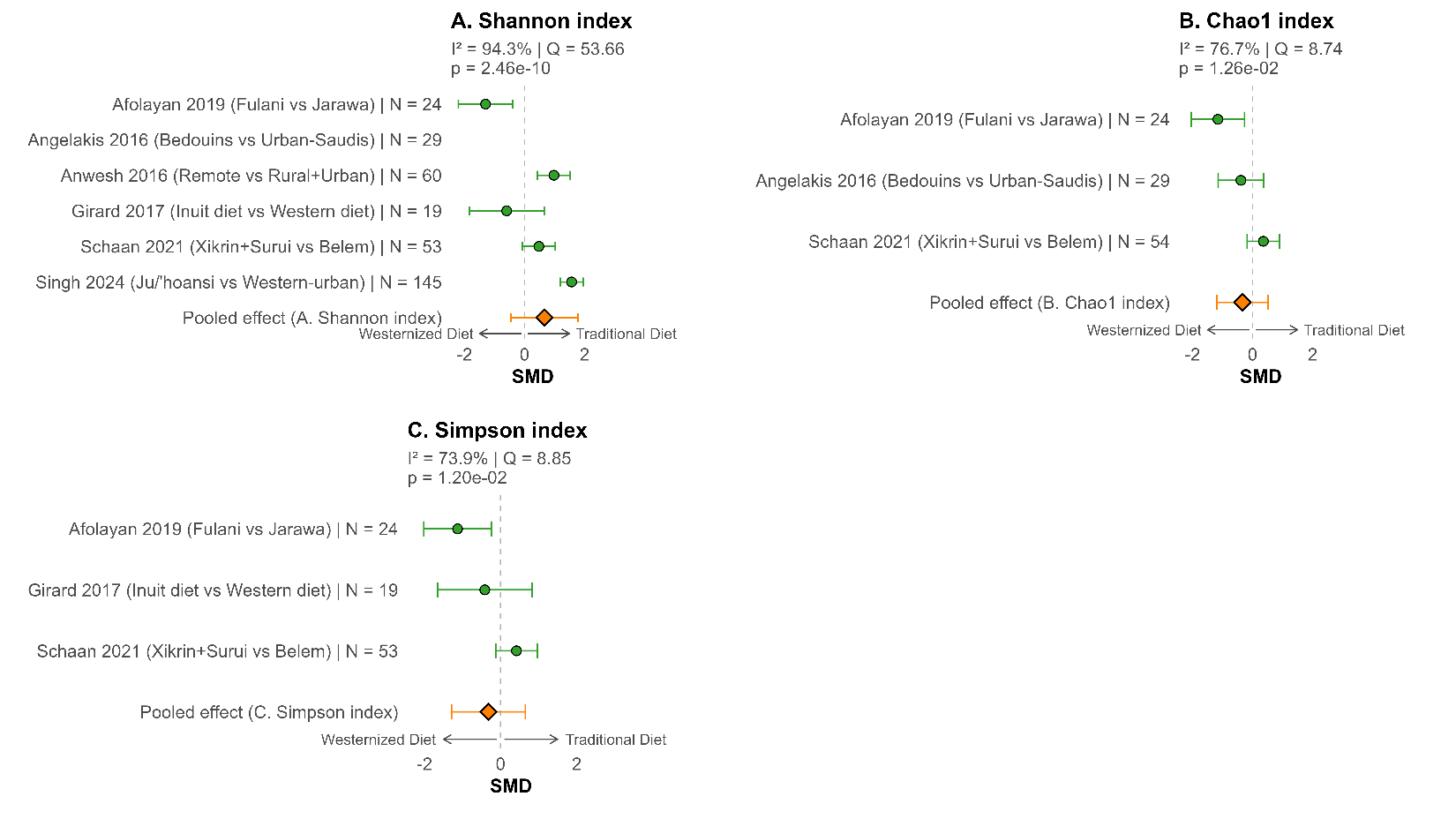


**Supplementary Figure 3. Gut microbiota diversity differences associated with dietary westernization in Indigenous populations: meta-analysis excluding Zhang (2).** Forest plots showing SMD in alpha-diversity indices—(A) Shannon, (B) Chao1, (C) Simpson—between Indigenous adults adhering to traditional versus westernized dietary patterns. The study conducted in Mongolia among nomadic pastoralists not officially recognized as Indigenous was excluded from this analysis. Positive SMD values indicate higher diversity under traditional diets. Pooled estimates (orange diamonds) and study weights are shown. I² values represent the percentage of total variation across studies due to heterogeneity rather than chance, with higher values (>75%) indicating considerable heterogeneity. SMD, standardized mean difference; I², heterogeneity; Q, Cochran’s Q; N, sample size.


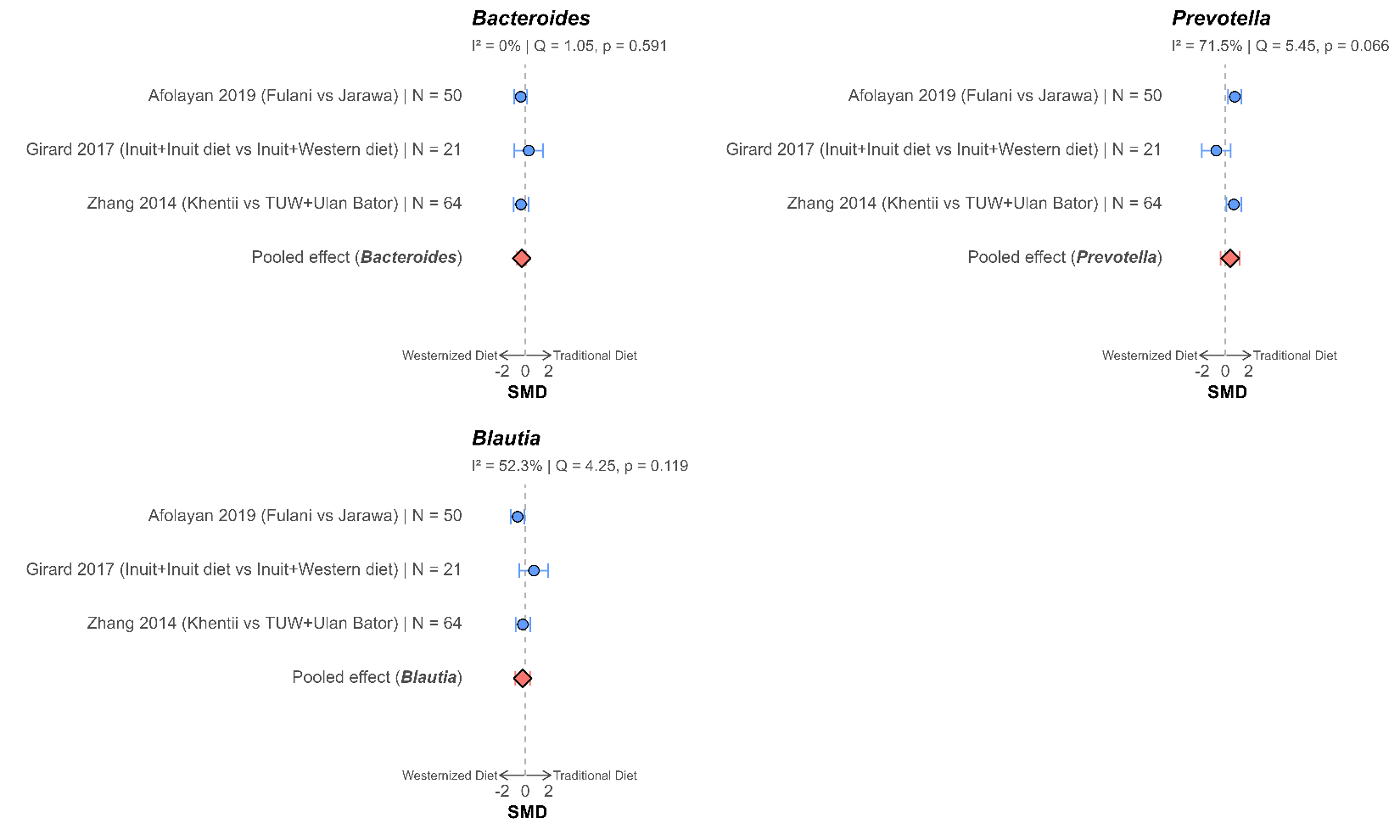


**Supplementary Figure 4. Differences in bacterial genera abundance between traditional and westernized diets: meta-analysis.** Forest plots showing SMD in the relative abundance of three dominant gut bacterial genera—*Bacteroides, Prevotella*, and *Blautia*—between population groups following traditional versus more westernized dietary patterns, across three independent studies (Girard (1), Zhang (2), Afolayan (7)). Positive SMD values indicate higher abundance under traditional diets. Pooled estimates (red diamonds) and study weights are shown. Each meta‑analysis used a random‑effects model with the REML estimator. I² values represent the percentage of total variation across studies due to heterogeneity rather than chance, with higher values (>75%) indicating considerable heterogeneity. SMD, standardized mean difference; I², heterogeneity; Q, Cochran’s Q; N, sample size.

*
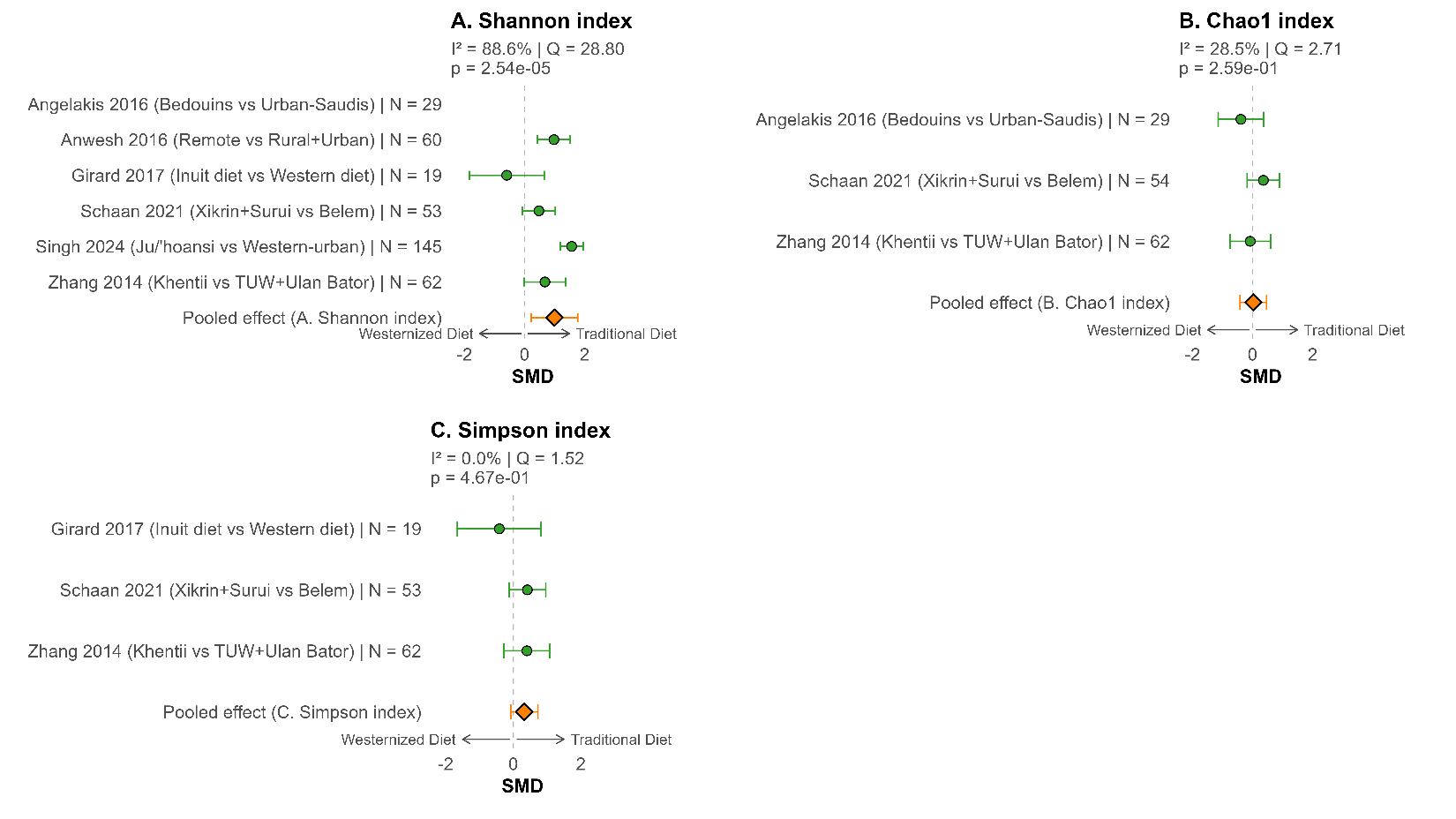
*

**Supplementary Figure 5. Gut microbiota diversity shifts associated with dietary westernization in Indigenous populations: meta-analysis excluding Afolayan (7).** Forest plots showing SMD in alpha-diversity indices—(A) Shannon, (B) Chao1, (C) Simpson—between Indigenous adults adhering to traditional versus more westernized dietary patterns. Positive SMD values indicate higher diversity under traditional diets. Pooled estimates (orange diamonds) and study weights are shown. Each meta‑analysis used a random‑effects model with the REML estimator. I² values represent the percentage of total variation across studies due to heterogeneity rather than chance, with higher values (>75%) indicating considerable heterogeneity. SMD, standardized mean difference; I², heterogeneity; Q, Cochran’s Q; N, sample size.

*
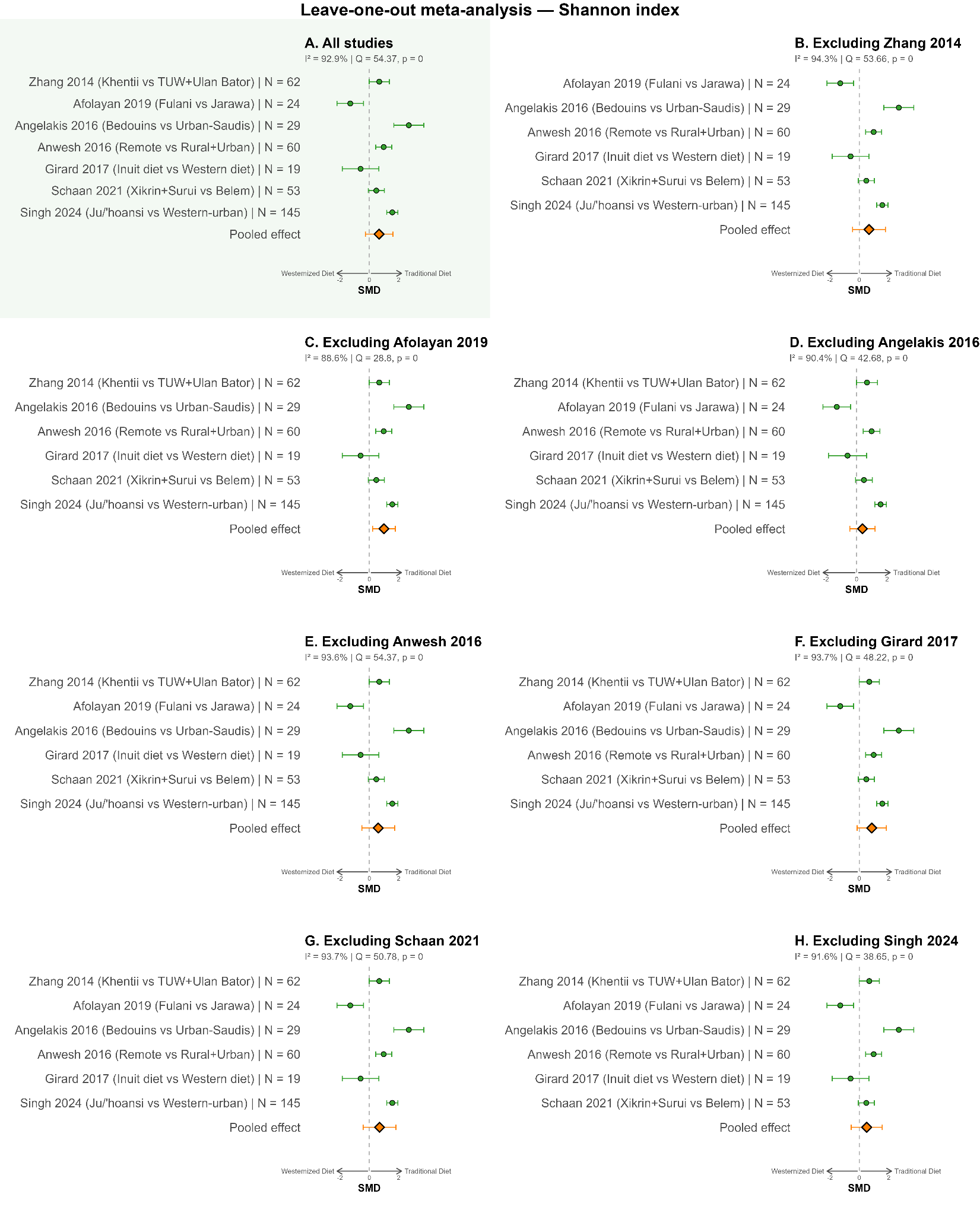
*

**Supplementary Figure 6.** **Leave-one-out sensitivity analysis of gut microbiota α-diversity (Shannon index).** Forest plots showing the impact of omitting each individual study on the pooled SMD in Shannon diversity between traditional and westernized dietary groups. Panel A includes all studies; Panels B–H show results when each indicated study is excluded in turn. Positive SMD values indicate higher microbial diversity in traditional-diet groups. SMD, standardized mean difference; I², heterogeneity; Q, Cochran’s Q; N, sample size.

*
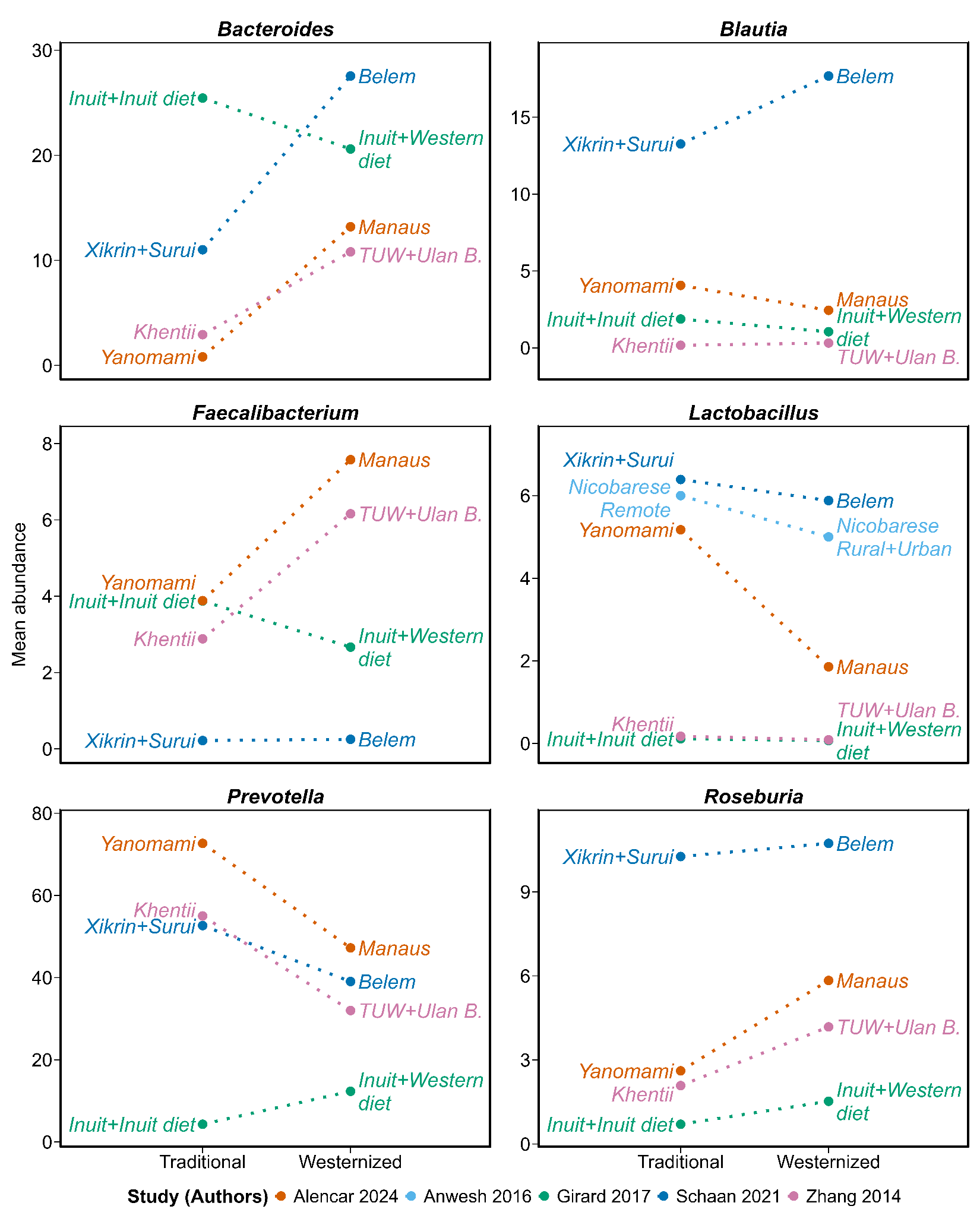
*

**Supplementary Figure 7. Genus-level differences associated with dietary westernization in Indigenous populations.** Slopegraphs showing the relative mean abundance of six dominant bacterial genera—*Bacteroides, Blautia, Faecalibacterium, Lactobacillus, Prevotella,* and R*oseburia*—in traditional versus westernized groups across five independent studies (Alencar 2024, Anwesh 2016, Schaan 2021, Girard 2017, Zhang 2014). Line colors differentiate studies. Points represent study-specific group means, and labels indicate the original group names used in each publication. Dotted lines are used to emphasize that values represent independent groups (traditional vs. westernized), and not repeated measurements within the same group (i.e., not longitudinal changes).

***
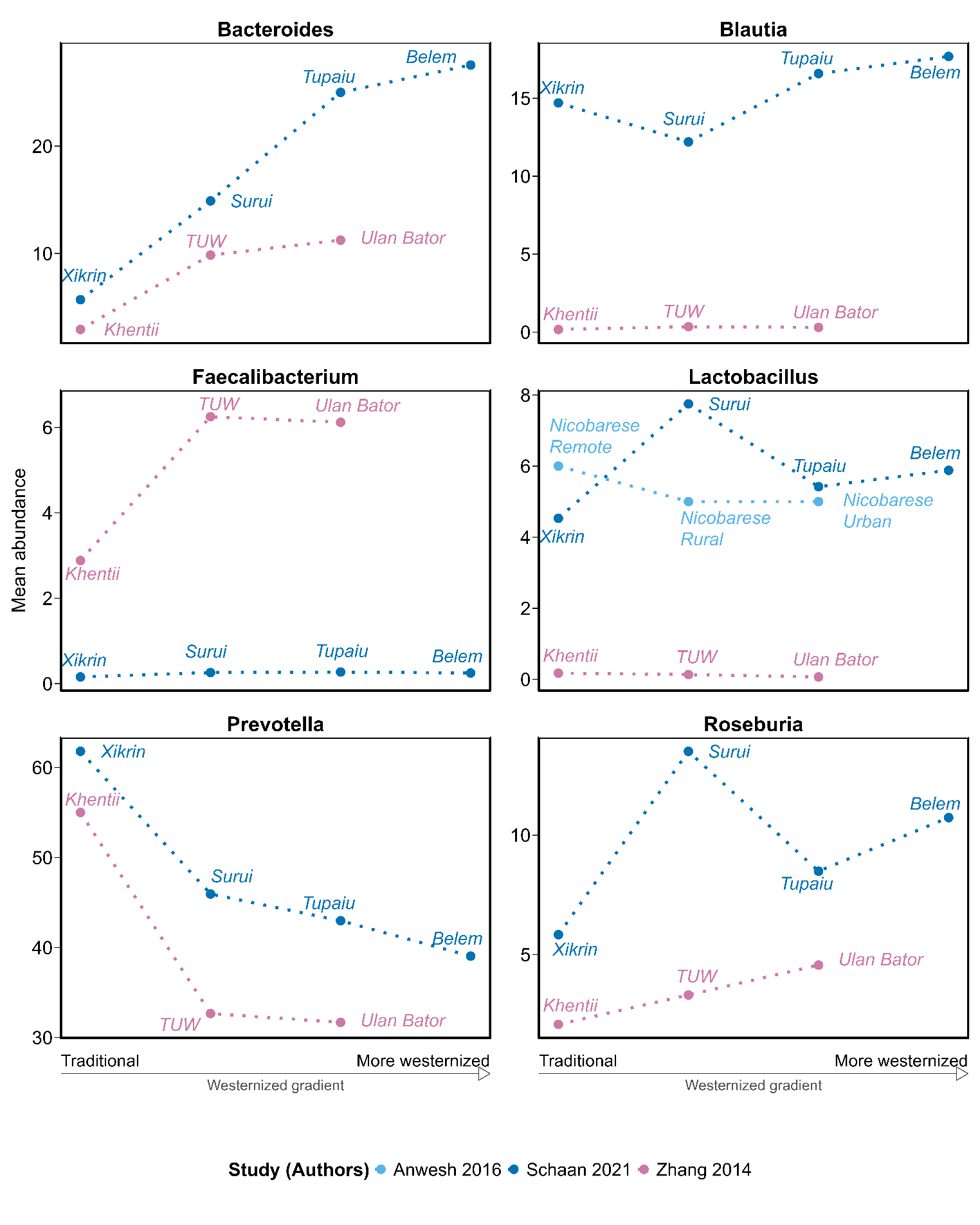
***

**Supplementary Figure 8. Mean abundance of dominant bacterial genera across an urbanization gradient.** Slopegraphs showing the relative mean abundance of six key bacterial genera—*Bacteroides, Blautia, Faecalibacterium, Lactobacillus, Prevotella*, and *Roseburia*—across groups varying in degree of urbanization, as reported in three independent studies (Zhang (2), Schaan (3), Anwesh (4)). Line colors differentiate studies. Points represent group-specific mean values, with labels showing the population names used in the original publications. Arrows indicate the presumed urbanization gradient from traditional to more urbanized contexts. Dotted lines are used to emphasize that values represent independent groups (traditional vs. westernized), and not repeated measurements within the same group (i.e., not longitudinal changes).

**References**

1. Girard C, Tromas N, Amyot M, Shapiro BJ. Gut Microbiome of the Canadian Arctic Inuit. Krajmalnik-Brown R, editor. mSphere. 2017 Feb 22;2(1):e00297-16.

2. Zhang J, Guo Z, Lim AAQ, Zheng Y, Koh EY, Ho D, et al. Mongolians core gut microbiota and its correlation with seasonal dietary changes. Sci Rep. 2014 May 16;4(1):5001.

3. Schaan AP, Sarquis D, Cavalcante GC, Magalhães L, Sacuena ERP, Costa J, et al. The structure of Brazilian Amazonian gut microbiomes in the process of urbanisation. npj Biofilms Microbiomes. 2021 Aug 5;7(1):65.

4. Anwesh M, Kumar KV, Nagarajan M, Chander MP, Kartick C, Paluru V. Elucidating the richness of bacterial groups in the gut of Nicobarese tribal community – Perspective on their lifestyle transition. Anaerobe. 2016 June;39:68–76.

5. Angelakis E, Yasir M, Bachar D, Azhar EI, Lagier JC, Bibi F, et al. Gut microbiome and dietary patterns in different Saudi populations and monkeys. Sci Rep. 2016 Aug 31;6(1):32191.

6. Singh M, Raina S, Goswami S, Raj D. Are the tribal highlanders protected from hypertension? A meta-analysis on prevalence of hypertension among high altitude tribal population of India. Indian J Public Health. 2020;64(3):295.

7. Afolayan AO, Ayeni FA, Moissl-Eichinger C, Gorkiewicz G, Halwachs B, Högenauer C. Impact of a Nomadic Pastoral Lifestyle on the Gut Microbiome in the Fulani Living in Nigeria. Front Microbiol. 2019 Sept 13;10:2138.

8. Alencar RM, Martínez JG, Machado VN, Alzate JF, Ortiz-Ojeda CP, Matias RR, et al. Preliminary profile of the gut microbiota from amerindians in the Brazilian amazon experiencing a process of transition to urbanization. Braz J Microbiol. 2024 Sept;55(3):2345–54.
